# Identification and heterologous expression of an NRPS biosynthetic gene cluster responsible for the production of the pyrazinones Ichizinone A, B and C

**DOI:** 10.1186/s12934-025-02753-6

**Published:** 2025-06-07

**Authors:** Patrick Oberhäuser, Maksym Myronovskyi, Marc Stierhof, Oleksandr Gromyko, Andriy Luzhetskyy

**Affiliations:** 1 Department of Pharmaceutical Biotechnology, Saarland University, 66123 Saarbrücken, Germany; 2 INM– Leibniz Institute for New Materials, Campus D2 2, 66123 Saarbrücken, Germany; 3 Department of Genetics and Biotechnology, Ivan Franko National University of Lviv, Lviv, 79005 Ukraine; 4 Microbial Culture Collection of Antibiotic Producers, Ivan Franko National University of Lviv, Lviv, 79005 Ukraine; 5 Helmholtz Institute for Pharmaceutical Research Saarland, 66123 Saarbrücken, Germany

**Keywords:** Actinobacteria, *Streptomyces*, Pyrazinone, NRPS, Heterologous expression, Gene cluster, Biosynthesis

## Abstract

Pyrazinones are a growing family of microbial NRPS-derived natural products showing interesting biological activities. These compounds are characterized by the presence of either a di- or trisubstituted heterocyclic, nonaromatic 2(1 H)-pyrazinone core in their structure. The most commonly occurring disubstituted pyrazinone natural products are synthesized through a dipeptide intermediate, which is further cyclized to yield the pyrazinone moiety. Trisubstituted pyrazinones are seldom found in natural products, with JBIR56 and JBIR57, isolated from marine *Streptomyces*, being notable examples. In contrast to the simply organized disubstituted pyrazinones, JBIR56 and JBIR57 are syn-thesized as tetrapeptides with unnatural beta-amino acid residue involved in the for-mation of the pyrazinone moiety. Despite interesting structural features, biosynthetic routes leading to the production of these compounds have not been reported yet. Here we report the discovery of new members of trisubstituted pyrazinone family– tetrapeptides ichizinones A-C in *Streptomyces* sp. LV45-129. Through sequence analysis and heterologous expression, a biosynthetic gene cluster encoding ichizinone production was identified. Based on gene annotation and sequence homology, a biosynthetic model was suggested. The presented results provide insights into the biosynthesis of rare trisubstituted pyrazinone natural products.

**Supplementary Information:**

The online version contains supplementary material available at 10.1186/s12934-025-02753-6.

## Introduction

**Fig. 1:**
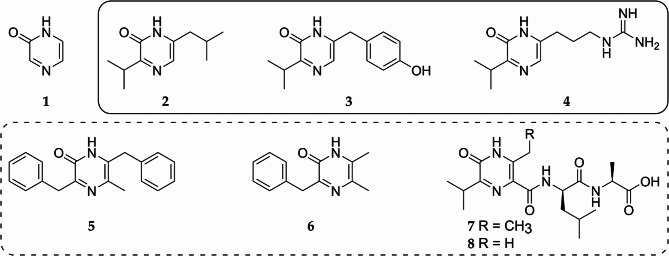
Representatives of the pyrazinone family of natural products: 1–2(1 H)-pyrazinone core structure (**1**), 2– leuvalin (**2**), 3– tyrvalin (**3**), 4– argvalin (**4**), 5– sorazinone B (**5**), 6– butrepyrazinone (**6**), 7– JBIR-56 (**7**), 8– JBIR-57 (**8**). The disubstituted pyrazinone natural products are enclosed within a solid-line rectangle, while the trisubstituted pyrazinone natural products are enclosed within a dashed-line rectangle

Pyrazinones represent a small family of natural products structurally related to diketopiperazines [1, 2]. The hallmark of these compounds is the six-membered heterocyclic 2(1 H)-pyrazinone ring (**1**) in their structure (Fig. 1) [1]. Pyrazinones are widely distributed across various domains of life, including fungi, staphylococci, streptomycetes, myxobacteria, and several marine sponge species [3–12]. From a structural perspective, pyrazinones can be classified into two subgroups: the more commonly occurring disubstituted 2(1 H)-pyrazinones and the less frequently found trisubstituted pyrazinones [7, 8, 13]. The disubstituted compounds include deoxyaspergillic acid and flavacol isolated from *Aspergillus flavus*, leuvalin (**2**), phevalin, and tyrvalin (**3**) from *Staphylococcus* species, phileucin, arglecin, and argvalin (**4**) from *Streptomyces*, as well as dragmacidin D and ma’edamines A and B from deep-water marine sponge species [2–4, 9, 11, 12, 14, 15] (Fig. 1). The trisubstituted pyrazinones include enhypyrazinones A and B, coralinones A and B, and sorazinone B (**5**) from various myxobacteria, butrepyrazinone (**6**) from *Verrucosispora* sp. K51G, and JBIR-56 (**7**) and JBIR-57 (**8**) from *Streptomyces* sp. SpD081030SC-03 [5–8, 10] (Fig. 1).

Biosynthetically, the majority of pyrazinones are condensation products of two amino acids. Typically, a dimodular nonribosomal peptide synthetase (NRPS) is involved in synthesizing the dipeptide precursor, which cyclizes upon release to form the pyrazinone moiety [2, 6, 11]. However, in contrast to the widespread, simply organized dipeptide-derived compounds, the pyrazinone family also includes more complexly synthesized members. For example, JBIR-56 (**7**) and JBIR-57 (**8**) are assembled from four amino acid residues. An unnatural beta-amino acid appears to play a role in forming the pyrazinone moiety of these compounds, which is further modified by attachment to a dipeptide chain [8]. Unfortunately, despite their intriguing structural features, the biosynthesis of JBIR-56 (**7**) and JBIR-57 (**8**) has not yet been elucidated.

In this study, we report the discovery of three new members of the trisubstituted pyrazinone family: ichizinones A (**9**), B (**10**), and C (**11**). These compounds were identified.

in extracts of *Streptomyces* sp. LV45-129 and are structurally closely related to JBIR-56 (**7**) and JBIR-57 (**8**). The ichizinones were isolated, and their structures were elucidated using nuclear magnetic resonance spectroscopy, MS/MS analysis, and Marfey’s analysis. To identify the biosynthetic gene cluster encoding the production of these compounds, we performed bioinformatics analysis of the genome sequence of the producing strain and confirmed the findings through heterologous expression in *Streptomyces albus* Del14. DNA deletion experiments and sequence analysis enabled us to propose a biosynthetic pathway leading to ichizinone production.

## Materials and methods

### General experimental procedures

All strains and plasmids used in this study are listed in Tables S1– S2. *Escherichia coli* strains were cultured in Luria-Bertani (LB) medium [16]. *Streptomyces* strains were cultivated on soya flour mannitol agar (MS agar) [17] for sporulation and conjugation and in liquid tryptic soy broth (TSB; Sigma-Aldrich, St. Louis, MO, USA). Liquid DNPM medium (40 g/L dextrin, 7.5 g/L soytone, 5 g/L baking yeast, and 21 g/L MOPS, pH 6.8) was used for cluster expression and secondary metabolite production. When required, the antibiotics kanamycin, apramycin, hygromycin and nalidixic acid were supplemented.

### DNA manipulations

Isolation of cosmids from the constructed genomic library of *S.* sp. LV45-129, DNA manipulations, transformation into *E. coli* and intergeneric conjugation between *E. coli* and *Streptomyces* were performed according to standard protocols [16–18]. Purification of cosmids was done by using the BACMAX ^TM^ DNA purification kit (Lucigen, Middleton, WI, USA). All restriction endonucleases were used according to manufacturer’s recommendations (New England Biolabs, Ipswich, MA, USA).

To determine the involvement of certain genes in the ichizinone biosynthesis, cosmids with the deletion of these genes were constructed. In the cosmid E514_KOA the cyclase gene was inactivated. In the cosmid E5124_KOB, the thioesterase gene was inactivated and in cosmid E514_KOC, the PKS gene was inactivated. For this purpose, the hygromycin cassette was amplified from the pACS-hyg plasmid with the pairs of primers E514_KOA_F/E514_KOA_R, E514_KOB_F/E514_KOB_R, E514_KOC_F/E514_KOC_R (Table S3). The obtained PCR fragments were utilized for the construction of the abovementioned cosmids using Red-ET modification [19]. The construction was confirmed using restriction mapping, PCR with the pairs of primers E514_KOA_chkF/E514_KOA_chkR, E514_KOB_chkF/E514_KOB_chkR, E514_KOC_chkF/E514_KOC_chkR (Table S3) and sequencing.

### Metabolite extraction and analysis

All *Streptomyces* strains were precultured in 25 mL of TSB for 24 h before 1 mL of each seed culture was used to inoculate 100 mL of DNPM production medium. Cultures were incubated at 28 °C for seven days. Metabolites were extracted with equal amounts of butanol from the supernatant of the culture broth, evaporated and dissolved in methanol. HPLC-MS analysis was performed by separating 1 µL of the extract using a Dionex UltiMate 3000 UPLC (Thermo Fisher Scientific, Waltham, MA, USA), a 10-cm ACQUITY UPLC BEH C18 column, 1.7 μm (Waters, Milford, MA, USA) and a linear gradient of 0.1% formic acid solution in acetonitrile against 0.1% formic acid solution in water from 5 to 95% in 18 min at a flow rate of 0.6 mL/min. Samples were analyzed using an amaZon speed mass spectrometer or maXis high resolution QTOF system (Bruker, USA). Data were collected and analyzed with the Bruker Compass Data Analysis software, version 4.1 (Bruker, Billerica, MA, USA). The monoisotopic mass was searched in the Dictionary of Natural Products database.

### Ichizinone isolation

For preparative metabolite isolation *Streptomyces* strains were precultured in 6 Flasks each containing 25 mL of TSB for 24 h before 100 flasks each containing 100 mL of DNPM were inoculated with 1 mL of the seed culture. Cultures were incubated for 7 days at 28 °C. The mycelial part was separated by centrifugation and metabolites were extracted from the supernatant as described above. The ichizinones were purified using size-exclusion and subsequent reverse phase chromatography. Size-exclusion chromatography was performed by using a Sephadex LH-20 column (GE Healthcare, USA) and methanol as a solvent. Fractions containing ichizinones were detected by LC-MS analysis, pooled together, evaporated and dissolved in methanol. Subsequently, preparative HPLC was performed on a Waters Autopurification system (Waters, Milford, MA, USA) equipped with a Waters 2545 Binary Gradient module using a Nucleodur C18 HTec column (5 μm, 250 × 21 mm, Macherey-Nagel, Düren, Germany). UV spectra were recorded with a photodiode array detector (Waters 2998, Waters, Milford, MA, USA). A composition of 0.1% formic acid containing water and methanol was used as a mobile phase. Individual peaks were collected and analyzed by LC-MS as described above. Finally, all collected peaks were purified by semipreparative HPLC (Dionex UltiMate 3000, Thermo Fisher Scientific, USA) using a C18 column (Synergi 10 μm, 250 × 10 mm; Phenomenex, Aschaffenburg, Germany).

**Fig. 2:**
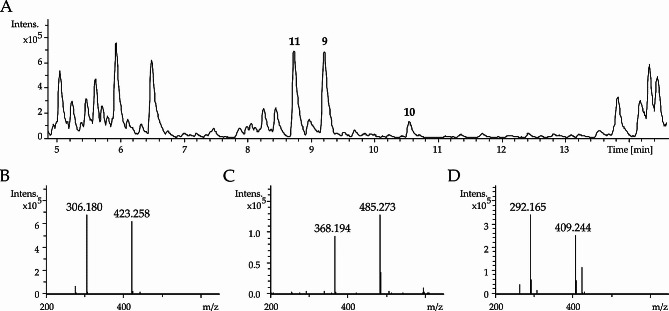
LC-MS detection of ichizinones. A– Base peak chromatogram of crude extract from *S.* sp. *LV45-129*. Peaks corresponding to ichizinones **A** (**9**), B (**10**), and **C** (**11**) are marked with the numbers 9, 10 and 11, respectively. **B**, **C**, and **D**– Mass spectra of the peaks corresponding to ichizinones **A** (**9**), **B** (**10**), and **C** (**11**), respectively

Ichizinone A (**9**): White powder; 1.4 mg; [α]D20 -25 (c 0.10, MeOH); UV (ACN/ H2O + 0.1% FA) λmax 226, 266 and 316 nm; NMR data, see Table 1; ESI-TOF-MS m/z 423.2597 [M + H]+ (calc. for C21H35N4O5 423.2602), see Fig. 2.

Ichizinone B (**10**): White powder; 1.0 mg; [α]D20 -20 (c 0.02, MeOH); UV data not available; NMR data, see Table S4; ESI-TOF-MS m/z 423.2597 [M + H]+ (calc. for C21H35N4O5 423.2602), see Fig. 2.

Ichizinone C (**11**): White powder; 4.4 mg; [α]D20 -22 (c 0.44, MeOH); UV (ACN/ H2O + 0.1% FA) λmax 226, 266 and 316 nm; NMR data, see Table S5; ESI-TOF-MS m/z 485.2752 [M + H]+ (calc. for C26H37N4O5 485.2758), see Fig. 2.

### NMR data acquisition and optical rotation

The chemical structures of all the compounds were determined via multidimensional NMR analysis. ^1^H-NMR, ^13^C-NMR, and 2D spectra were recorded either at 700 MHz (1H)/175 MHz (^13^C) ichizinone A (**9**) or at 500 MHz (1H)/126 MHz (^13^C) for ichizinones B (**10**) and C (**11**). Experiments were conducted in the Ascend 700 spectrometer using a cryogenically cooled triple resonance probe (Bruker BioSpin, Rheinstetten, Germany) or in the Bruker Avance Neo 500 MHz, equipped with a Prodigy Cryo-probe. Samples were dissolved in methanol-d_3_ or dimethyl sulfoxide-d_6_. Chemical shifts are reported in ppm relative to tetramethylsilane; the solvent was used as the internal standard. Coupling constants are reported in Hertz (Hz). Multiplicity is reported with the usual abbreviations (s: singlet, br s: broad singlet, d: doublet, dd: doublet of doublets, t: triplet, dt: doublet of triplets, q: quartet, dq: doublet of quartets, m: multiplet).

Optical rotations were measured using a JASCO P-2000 digital polarimeter (28600 Mary’s Ct, Easton, MD, USA).

### Marfey’s method

200 µg of ichizinone C (**11**) was hydrolyzed in 100 µL 6 N HCl at 100 °C for 45 min in a closed vial filled with nitrogen. The sample was then dried for 15 min and dissolved in 110 µL of water before 50 µL were transferred to a 1.5 mL Eppendorf tube. Subsequently, 20 µL of 1 N NaHCO_3_ and 20 µL of 1% L-FDLA or D-FDLA in acetone were added to the hydrolysate [20]. The amino acid standards were prepared in the same way using only L-FDLA. All reaction mixtures were incubated at 40 °C for 2 h at 700 rpm and subsequently quenched with 10 µL 2 N HCL to stop the reaction. The samples were diluted with 300 µL ACN to a total volume of 400 µL, from which 1 µL of each sample was analyzed with a maXis high-resolution LC-QTOF system using aqueous ACN with 0.1% FA and an adjusted gradient of 5–10% for 2 min, 10–25% for 13 min, 25–50% for 7 min and 50–95% for 2 min. Sample detection was carried out at 340 nm.

### Genome mining and bioinformatics analysis

The *S.* sp. LV45-129 genome was screened for secondary metabolite biosynthetic gene clusters using the antiSMASH online tool [21]. Genetic data was analyzed with the software Geneious prime 2022.2.2 [22]. Selected genes were further analyzed using the BLAST online tool from the National Center for Biotechnology Information (http://www.ncbi.nlm.nih.gov/BLAST/) and the Universal Protein Resource (UniProt) (https://www.uniprot.org/blast).

### Antimicrobial susceptibility test

Minimum inhibitory concentrations (MICs) were determined according to standard procedures. Single colonies of the tested strains were suspended in cation-adjusted Müller-Hinton broth to achieve a final inoculum of approximately 104 CFU mL^− 1^. Serial dilutions of 409 (0.03 to 64 µg mL^− 1^) were prepared in sterile 96-well plates before the strain suspension was added. Growth inhibition was assessed after overnight incubation (16–18 h) at 30–37 °C. A panel consisting of the following strains was tested: *B. subtilis* DSM-10, *S. aureus* Newman, *Mycobacterium smegmatis* MC2155, *Citrobacter freundii* DSM-30,039, *E. coli* BW25113 (wt), *E. coli* JW0451-2 (∆acrB), *Pseudomonas aeruginosa* PA14 DSM-19,882, Acinetobacter baumanii DSM-30,008, *Mucor hiemalis* DSM-2656, *Pichia anomala DSM-6766*, *Cryptococcus neoformans* DSM-11,959, *Candida albicans* DSM-1665, CHO-K1 and HepG2.

## Results and discussion

### Identification and isolation of Ichizinones A, B and C in the culture broth of *streptomyces* Sp. LV45-129

The strain *S.* sp. LV45-129 was thoroughly investigated as part of a broader screening for new bioactive metabolites. For this purpose, the strain was fermented in the production medium DNPM. The culture broth was then extracted with butanol, and the resulting extract analyzed using high-resolution liquid chromatography-mass spectrometry (LC-MS). Besides the identification of known metabolites such as puromycin and pamamycin, three peaks were detected at the retention times of 8.8 min, 9.2 min and 10.6 min, corresponding to the compounds with [M + H]^+^ 409.244 m/z, [M + H]^+^ 423.258 m/z and [M + H]^+^ 485.273 m/z respectively (Fig. 2, Fig. S1). A search in the natural product database for these high-resolution masses did not generate any matches, implying that the identified compounds might be new.

In order to isolate the identified compounds, we performed a large scale cultivation of the strain *S.* sp. LV45-129. The strain was inoculated into 10 L of DNPM production medium, and the culture broth was subsequently extracted with butanol. The compounds were first separated from contaminants using size-exclusion chromatography and then further purified through preparative reverse-phase chromatography. With this procedure, we obtained 1.4 mg of the compound with [M + H]^+^ 423.258 m/z, 1 mg of the compound with [M + H]^+^ 485.273 m/z, and 4.4 mg of the compound with [M + H]^+^ 409.244. The isolated compounds were named ichizinone A (**9**), B (**10**) and C (**11**), respectively. To gain insights into the structure of the ichizinones, the purified compounds were used for the structure elucidation experiments. Additionally, the isolated compounds were subjected to biological activity studies to assess their potential antimicrobial properties. The ichizinones showed no activity against the tested Gram-positive and Gram-negative bacteria or the fungal strains.

### Structure Elucidation of Ichizinone A, B and C

**Fig. 3:**
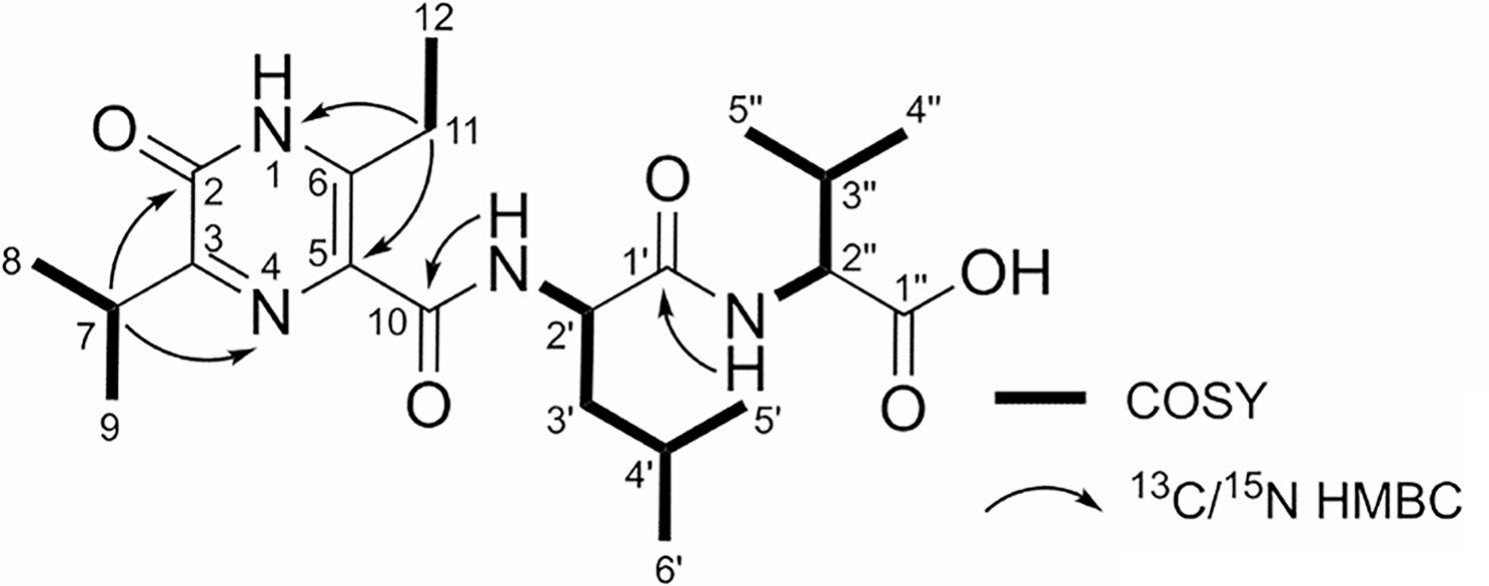
^13^C/^15^N HMBC () and COSY (s) key correlations of ichizinone A

The molecular formula of ichizinone A (**9**) was determined to be C_21_H_34_N_4_O_5_ based on HRMS data ([M + H]^+^, m/z 423.258), indicating that the compound is a peptide. The structure of ichizinone A (**9**) was elucidated using 1D and 2D NMR experiments and corroborated by MS fragmentation data (Table 1, Fig. 3, Fig. S2–S9, S26). For NMR analysis, the compound was thoroughly dried and measured in DMSO and DMSO containing traces of TFA to achieve full protonation of NH groups. The latter resulted in sharper signals due to reduced proton exchange. The data quality generally improved upon the addition of TFA, however, two carbon signals at δ_H_ 122.59 and δ_H_ 157.85 vanished and could only be observed in TFA free DMSO (Fig. S9). The amino signals at δ_H_ 8.23 (2’-NH) and δ_H_ 8.41 (2‘’-NH) showing COSY correlations to the methines H-2‘ (δ_H_ 4.69) and H-2‘‘ (δ_H_ 4.17) indicated the presence of two amino acids. Analyzing the spin system starting from the α-methine proton at H-2‘ together and the aliphatic proton signals at δ_H_ 1.53 (CH_2_), 1.57 (CH), 0.92 (CH_3_) and 0.89 (CH_3_) revealed the amino acid leucine. The spin system starting from the α-methine proton at H-2‘‘, followed by δ_H_ 2.07 (CH), 0.86 (CH_3_) and 0.87 (CH_3_) was assigned to valine. An HMBC correlation of the carbonyl C-1‘ (δ_C_ 172.59) of leucine to 2’’-NH of valine showed that the two amino acids are connected via a peptide bond, while the carbonyl group of valine did not show any correlation, revealing a carboxylic acid terminus. The remaining two spin systems were assigned based on COSY correlations of H-7, H-8 and H-9 revealing an isopropyl group and H-11 and H-12 revealing an ethyl group. HMBC correlations of H-11 to C-6 (δ_C_ 146.09), C-5 (δ_C_ 122.59) and N-1 (δ_N_ 175.38), and H-7(δ_H_ 1.16) to C-2 (δ_C_ 157.85), C-3 (δ_C_ 158.92) and N-4 (δ_N_ 317.96) suggests that the isopropyl and the ethyl group are attached to a pyrazinone heterocycle. A database search of the suggested final structure elements revealed strong similarities to compound JBIR-56 (**7**) [8]. Correlations in the ^1^H-^13^CHMBC from 1-NH to 2-C or 3-C and in the ^15^N-HMBC to either 1-NH or 4 N could not be observed. However, alignment of ^1^H and ^13^C shifts of ichizinone A (**9**) and JBIR-56 (**7**) and analysis of MS/MS fragmentation patterns (Fig. S26) strongly indicated the structural moieties are arranged in a pyrazinone moiety (Fig. 3; Table 1). The elucidated compound ichizinone A (**9**) was identified as a novel natural compound.

**Table 1:** NMR data of Ichizinone A (**9**)

Ichizinone A (9)					JBIR-56 (7)	
No, type	δ(13 C, 15 N) [ppm]	δ(1 H) [ppm], mult(J)	COSY (H-)	HMBC (C-/N-)	δ(13 C, 15 N) [ppm]	δ(1 H) [ppm], mult(J)
1-NH	175.38			11		
2-C*	157.85*			7*	155.9	
3-C	158.92			7, 8, 9	158.7	
4-N	317.96			7		
5-C*	122.59*			11*	120.3	
6-C	146.09			11, 12	145.9	
7-CH	29.94	3.23, m	8, 9	8, 9	29.5	3.22, q (6.6)
8-CH3	20.25	1.16, ovl**	7	7, 9	19.9	1.15, d (6.6)
9-CH3	20.33	1.16, ovl**	7	7, 8	19.8	1.15, d (6.6)
10-C	163.62			2’-NH	163.3	
11-CH2	23.35	3.02, m 2.93, m	12	12	23.2	2.99, dq (12.6, 7.2) 2.93, dq (12.6, 7.2)
12-CH3	14.27	1.13, t (7.5)	11	11	14.0	1.13, (7.2)
Leu					Leu	
1’-C	172.59			2’, 3’, 2’’-NH	171.4	
2’-CH	51.18	4.69, dt (7.5, 8.4)	3’, 2’-NH	3’, 2’-NH	50.7	4.52, dd (13.8, 8.4)
3’-CH2	43.10	1.53, m	2’, 4’	2’, 5’, 6’	42.6	1.52, dd (13.8, 6.0)
4’-CH	25.22	1.57, m	3’, 5’, 6’	2’, 3’, 5’, 6’	24.7	1.56, d (6.0)
5’-CH3	22.72	0.92, d (5.8)	4’	3’, 6’	23.1	0.89, d (6.0)
6’-CH3	23.66	0.89, d (5.8)	4’	3’, 5’	22.4	0.88, d (6.0)
2’-NH	113.98	8.23, d (8.7)	2’	3’		8.18, d (8.4)
Val					Ala	
1’’-C	173.43	-		2’’	174.0	
2’’-CH	57.52	4.17, dd (6.7, 8.7)	2’’-NH, 3’’	3’’, 4’’, 5’’, 2’’-NH	48.1	4.12, dq (7.2, 6.6)
3’’-CH	30.53	2.07, m	2’’, 4’’, 5’’	2’’, 4’’, 5’’	17.7	1.23, d (7.2)
4’’-CH3	18.34	0.86, ovl**	3’’	2’’, 3’’, 5’’		
5’’-CH3	19.90	0.87, ovl**	3’’	2’’, 3’’, 4’’		
2’’-NH	114.71	8.41, d (8.6)	2’’	3’’		8.34, br s

**Fig. 4:**
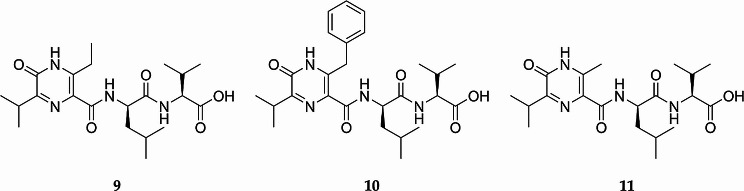
Structures of the isolated compounds: ichizinone **A** (**9**), ichizinone **B** (**10**), ichizinone **C** (**11**)

The molecular formula of ichizinone B (**10**) was determined as C_26_H_36_N_4_O_5_ based on the HRMS data ([M + H]^+^, m/z 485.273). The structure of ichizinone B (**10**), as shown in Fig. 4, was determined by 1D and 2D NMR experiments (Fig. S10–S17, S27, Table S4). The ^1^H-NMR spectrum revealed aromatic methines δH 7.34 (H-13/17), 7.25 (H-14/26) and 7.20 (H-15) which were assigned to a benzyl group. The benzyl group was found to be attached to the pyrazinone moiety at C-6, while the core structure of ichizinone B (**10**) is the same as that of ichizinone A (**9**). The structure of the compound was further confirmed through MS/MS fragmentation analysis. The observed fragmentation pattern of ichizinone B (**10**) closely corresponded to that of ichizinone A (**9**), with the only differences arising from the distinct substituents at position C-6 of the pyrazinone moiety (Fig. S26–S27). The elucidated compound ichizinone B (**10**) was identified as a novel natural compound.

The molecular formula of ichizinone C (**11**) was determined as C_20_H_32_N_4_O_5_ based on the HRMS data ([M + H]^+^, m/z 409.244). The structure of ichizinone C (**11**) (Fig. 4) was determined by 1D and 2D NMR experiments (Fig. S18–S25, S28, Table S5). This compound is structurally similar to other ichizinones, differing only by the presence of a methyl group attached to the pyrazinone moiety at C-6, instead of an ethyl or benzyl group as found in ichizinones A (**9**) and B (**10**), respectively. The absolute stereochemistry of the amino acids not incorporated in the pyrazinone moiety was determined for ichizinone C (**11**) using Marfey’s method [20], revealing L-valine and D-leucine as the constituent amino acids (Fig. S29).

### Identification of the Ichizinone gene cluster through heterologous expression in *streptomyces albus* Del14

**Fig. 5:**
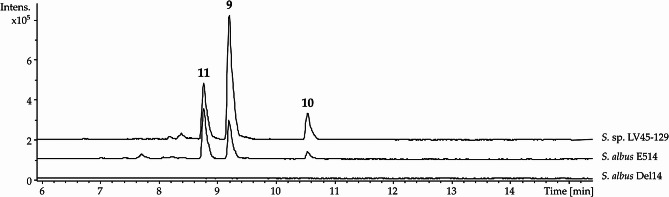
LC-MS analysis of ichizinone production by the heterologous host *S. albus* E514. Extracted ion chromatograms (409.3 ± 0.5 Da, 423.3 ± 0.5 Da, 485.3 ± 0.5 Da) of crude extracts from *S.* sp. LV45-129, *S. albus* E514, and *S. albus* Del14 are shown. Peaks corresponding to ichizinones **A** (**9**), **B** (**10**), and **C** (**11**) are marked with the numbers 9, 10, and 11, respectively

The isolated ichizinones are pyrazinones, each consisting of four amino acid residues. The pyrazinone moiety of ichizinones A (**9**), B (**10**), and C (**11**) is derived from a valine residue and a beta-amino acid residue, which varies among the isolated compounds: 3-amino-pentanoic acid, 3-amino-4-phenylbutanoic acid, and 3-aminobutanoic acid, respectively. The free carboxyl group of the beta-amino acid is extended by the attachment of a dipeptide composed of leucine and valine residues. The pyrazinone moiety of natural products is typically assembled by a small dimodular nonribosomal peptide synthetase [2, 6, 11]. However, the tetrapeptide nature of ichizinones suggests the involvement of a larger NRPS system composed of four modules. Genome sequence analysis of the ichizinone producer *S.* sp. LV45-129 using the antiSMASH software revealed 38 putative gene clusters associated with secondary metabolite production, including eight involved in the biosynthesis of NRPS-type products [21]. The detailed analysis of NRPS clusters identified one cluster as the most likely candidate for encoding ichizinone production. This cluster encodes a four-module NRPS with adenylation domains that show specificity for the amino acids valine, phenylalanine, and leucine, some of which are present in the ichizinone structures. To confirm that the identified cluster is responsible for ichizinone production, a genome library of the producer strain was constructed on the integrative cosmid vector cos15A_gus-AmInt and subsequently sequenced [23]. The cosmid E514, containing the entire NRPS cluster presumably involved in ichizinone production, was transferred into the heterologous host strain *Streptomyces albus* Del14 via conjugation [24]. The exconjugant strain *S. albus* E514 and the corresponding control strain, *S. albus* Del14, which lacks the cosmid, were fermented in DNPM production medium. The culture filtrates of both strains were extracted with butanol, and the resulting extracts were analyzed using LC-MS. This analysis identified ichizinones A (**9**), B (**10**), and C (**11**) in the extract of *S. albus* E514 (Fig. 5). The retention times and m/z values of the detected peaks were identical to those observed in the extract of the natural producer strain *S.* sp. LV45-129. In contrast, the extract of the control strain, *S. albus* Del14, showed no peaks corresponding to ichizinones. These results clearly demonstrate that the cosmid E514 contains the entire biosynthetic gene cluster for the production of ichizinones.

**Fig. 6:**
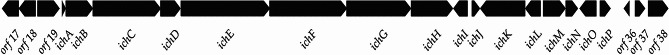
The chromosomal fragment of *S*. sp. LV45-129 containing the ichizonone biosynthetic gene cluster

The chromosome fragment cloned in the cosmid E514 spans a 52.6 kb DNA region and contains 40 genes (accession number PQ885478). Sequence analysis of this DNA fragment was conducted to define the borders of the ichizinone cluster. A set of 16 genes, *ichA*–*ichP*, was predicted to be involved in the biosynthesis of the compound (Table 2, Fig. 6). These genes encode four nonribosomal peptide synthetases, three regulatory proteins, three transport proteins, an MbtH-like protein, a cyclase, a thioesterase, a type I polyketide synthase, a monooxygenase, and a hypothetical protein. ClusterBlast analysis indicates that these genes are clustered together across various *Streptomyces* species, suggesting they constitute the ichizinone biosynthetic cluster. The 5’ upstream region of *ichA* contains genes encoding a Ku protein and two ATP-dependent DNA ligases– proteins involved in DNA repair mechanisms and not in ichizinone production [25, 26]. Similarly, the genes located downstream of *ichP* encode an arginyl-tRNA and an arginyl-tRNA synthetase, which are also unrelated to ichizinone biosynthesis. These findings further support the hypothesis that the genes *ichA*– *ichP* comprise the ichizinone biosynthetic cluster. However, since the inactivation of the genes flanking the *ichA–ichP* region was not performed, the possibility that additional genes are involved in ichizinone biosynthesis cannot be excluded.

**Table 2:** Proposed functions of genes within the Ichizinone gene cluster

Gene	Proposed Function	GeneBank homologue
*orf17*	ATP-dependent DNA ligase	WP_055529559.1
*orf18*	ATP-dependent DNA ligase	WP_055529557.1
*orf19*	KU protein	WP_055529555.1
*ichA*	MbtH family NRPS accessory protein	WP_150477341.1
*ichB*	MFS Transporter	WP_150477342.1
*ichC*	NRPS	WP_055529551.1
*ichD*	FMN-dependent luciferase-like monooxygenase	WP_055529549.1
*ichE*	NRPS	WP_167532726.1
*ichF*	NRPS	WP_150477345.1
*ichG*	NRPS	WP_150477346.1
*ichH*	TypeI Polyketide synthase	WP_150477347.1
*ichI*	Thioesterase	WP_055529989.1
*ichJ*	Polyketide cyclase	WP_055529992.1
*ichK*	ABC transporter permease	WP_055529993.1
*ichL*	ABC transporter ATP-binding protein	WP_055529995.1
*ichM*	Sensor histidine kinase	WP_055529997.1
*ichN*	Response regulator	WP_055530004.1
*ichO*	LysR family transcriptional regulator	WP_055530006.1
*ichP*	Hypothetical protein	WP_055530008.1
*orf36*	Arg tRNA	WP_246201615.1
*orf37*	Response regulator	WP_234336420.1
*orf38*	Arginyl-tRNA synthetase	WP_030792088.1

**Fig. 7:**
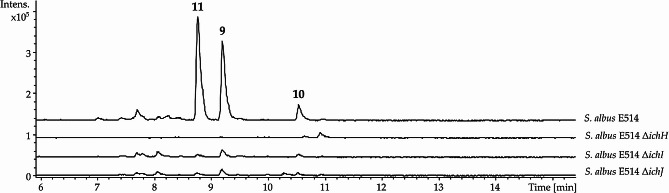
LC-MS analysis of ichizinone production in *S. albus* strains harboring E514 cosmids with inactivated biosynthetic genes. Extracted ion chromatograms (409.3 ± 0.5 Da, 423.3 ± 0.5 Da, 485.3 ± 0.5 Da) of crude extracts from *S. albus* E514, *S. albus* E514 Δ*ichH*, *S. albus* E514 Δ*ichI*, and *S. albus* E514 Δ*ichJ* are shown. Peaks corresponding to ichizinones **A** (**9**), **B** (**10**), and **C** (**11**) are marked with the numbers 9, 10, and 11, respectively

To confirm the involvement of *ichA– ichP* genes in ichizinone production, the genes *ichH*, *ichI*, and *ichJ*, which encode a type I polyketide synthase, a thioesterase, and a cyclase, respectively, were deleted in cosmid E514. The resulting recombinant constructs were expressed in the heterologous host *S. albus* J1074. Inactivation of the polyketide synthase led to a complete cessation of ichizinone production, while inactivation of the other two genes resulted in severely impaired production, with only trace amounts of the compounds being detected (Fig. 7). These findings corroborate that the identified set of genes is involved in ichizinone biosynthesis.

### Insights into the biosynthesis of Ichizinones

Ichizinones isolated in this study belong to the group of pyrazinone natural products. The most commonly occurring pyrazinones are small cyclic dipeptides composed of two proteinogenic amino acids [2–7, 11, 12, 15]. Their biosynthesis involves a dimodular NRPS that forms a dipeptide precursor, which is cyclized upon release to yield a disubstituted six-membered heterocyclic 2(1 H)-pyrazinone ring (**1**) [2, 11]. The substituents are positioned para to each other and correspond to the side chains of the amino acids involved in the ring formation (Fig. 1). In contrast to these dipeptide pyrazinones, the ichizinones produced by *S.* sp. LV45-129 are more complex (Fig. 4). These compounds are tetrapeptides composed of three proteinogenic amino acid residues and one nonproteinogenic beta-amino acid residue in the second position. The N-terminal amino acid residue and the second nonproteinogenic amino acid residue participate in forming the trisubstituted pyrazinone ring. The mechanism of pyrazinone ring formation of ichizinones appears to differ from that of disubstituted dipeptide pyrazinones. In the latter, ring closure is proposed to occur through the interaction of the free amino group of the N-terminal amino acid and the carboxyl group of the C-terminal amino acid [2, 11]. In ichizinones, however, the pyrazinone ring formation likely involves the interaction of the amino group of the N-terminal amino acid residue with the alpha carbon of the second nonproteinogenic beta-amino acid residue. Consequently, the carboxyl group of this beta-amino acid becomes the third substituent of the pyrazinone ring and serves as the attachment site for the remaining two amino acid residues (Fig. 4). As with disubstituted pyrazinones, the side chains of the amino acid residues involved in pyrazinone ring formation form the first and second substituents of the trisubstituted pyrazinone ring in ichizinones. Structurally, the isolated ichizinones strongly resemble compounds JBIR-56 (**7**) and JBIR-57 (**8**) (Fig. 1), which were identified in 2011 from a marine sponge-derived *Streptomyces sp.* SpD081030SC-03 [8]. However, the biosynthesis of these compounds has not been published.

**Fig. 8:**
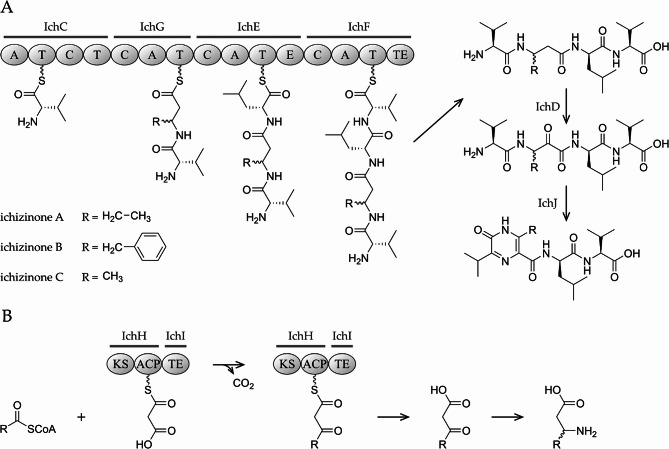
Proposed biosynthetic pathway of ichizinones. **A**– Assembly of ichizinones by linear NRPS. **B**– Biosynthesis of beta-amino acid precursors of ichizinones

The results of sequence analysis and heterologous expression experiments demonstrated that, as in the case of disubstituted pyrazinones, an NRPS is involved in the biosynthesis of ichizinones. The ichizinone biosynthetic cluster was predicted to comprise 16 genes, *ichA*–*ichP*, of which only eight encode structural biosynthetic enzymes. The genes *ichC*, *ichE*, *ichF*, and *ichG* encode individual modules of a four-module NRPS (Fig. 8), while *ichH* encodes a single-module type I polyketide synthase. The genes *ichD* and *ichI* encode a flavin-dependent oxidoreductase and a type II thioesterase, respectively. The product of the *ichJ* gene exhibits low sequence similarity to proteins of the SRPBCC family with uncharacterized functions and to members of the polyketide cyclase/dehydrase family. The closest characterized homologs of IchJ are the aromatase/cyclase proteins ZhuI and TcmN from polyketide biosynthetic pathways [27, 28]. The *ichA* gene encodes an MbtH family protein, which is often associated with bacterial NRPS. MbtH proteins are reported to bind noncovalently to the adenylation domains of some NRPS, thereby promoting their folding, stability, and activity [29, 30].

The number of modules in the NRPS encoded by *ichC*, *ichE*, *ichF*, and *ichG* aligns with the length of the ichizinone tetrapeptide precursor. At the first, third, and fourth positions, all ichizinones contain valine, leucine, and valine residues, respectively. Marfey’s analysis of the ichizinone C (**11**) hydrolysate demonstrated that the valine residue of its dipeptide tail is in the L-configuration, while the leucine residue is in the D-configuration. The isolated ichizinones differ in the nonproteinogenic amino acid residue at the second position: ichizinones A (**9**), B (**10**), and C (**11**) contain 3-aminopentanoic acid, 3-amino-4-phenylbutanoic acid, and 3-aminobutanoic acid, respectively. According to predictions made by antiSMASH and PKS/NRPS analysis bioinformatics tools, the.

adenylation domains of IchC, IchE, and IchF exhibit substrate specificity for valine, leucine, and valine, respectively– amino acids that are components of ichizinones [21, 31]. The NRPS module encoded by *ichE* contains an additional epimerization domain, which converts L-leucine to its D-stereoisomer. The substrate specificity of the adenylation domain of IchG could not be clearly predicted. We propose that the NRPS module encoded by *ichG* is responsible for the incorporation of various nonproteinogenic beta-amino acids found in the structures of ichizinones. According to the domain organization of NRPS modules encoded by *ichC*, *ichE*, *ichF*, and *ichG*, we propose that they form an NRPS assembly line in the following order: *ichC* encodes an initiation module responsible for the incorporation of the N-terminal L-valine residue (Fig. 8). The module encoded by *ichG* acts as the first elongation module, incorporating the nonproteinogenic amino acid. The second elongation module, encoded by *ichE*, incorporates L-leucine and converts it to the D-configuration. Finally, the termination NRPS module, encoded by *ichF*, incorporates the C-terminal L-valine residue. The thioesterase domain of IchG releases the tetrapeptide precursor, which is subsequently processed to yield the ichizinone molecule.

The origin of nonproteinogenic beta-amino acids is one of the most intriguing aspects of ichizinone biosynthesis but remains incompletely understood. We propose that the genes *ichH* and *ichI*, whose deletions result in the cessation of ichizinone production, are involved in the biosynthesis of these beta-amino acids. The *ichH* gene encodes a single-module type I PKS comprising only ketosynthase (KS) and acyl carrier protein (ACP) domains, while *ichI* encodes a type II thioesterase. According to our hypothesis, the PKS utilizes free fatty acyl-CoA esters: acetyl-CoA, propionyl-CoA, and phenylacetyl-CoA as starter units for decarboxylative condensation with malonate (Fig. 8). Acetyl-CoA and propionyl-CoA are common acyl-CoA esters present in bacterial cells. The detection of phenylacetic acid and phenylacetate-CoA ligase activity has also been reported in *Streptomyces* [32–34]. Since the PKS lacks a dedicated acyltransferase (AT) domain, we hypothesize that an unidentified trans-acyltransferase, encoded elsewhere in the host strain’s genome, transfers the malonyl moiety from malonyl-CoA to the ACP domain of IchH. The resulting diketides 3-oxobutanoic acid, 3-oxopentanoic acid, and 3-oxo-4-phenylbutanoic acid are released from the PKS by the action of the thioesterase IchI. We further propose that the resulting beta-keto acids are aminated at the beta position by an unidentified aminotransferase encoded in the host strain’s genome, yielding the beta-amino acids 3-aminobutanoic acid, 3-aminopentanoic acid, and 3-amino-4-phenylbutanoic acid.

To confirm the involvement of the PKS IchH in the biosynthesis of beta-amino acid precursors of ichizinones, the *S. albus* E514 Δ*ichH* strain harboring the ichizinone cluster with the inactivated gene *ichH* was supplemented with beta-amino acids: 3-aminobutanoic acid, 3-aminopentanoic acid, and 3-amino-4-phenylbutanoic acid. However, LC-MS analysis of the culture extracts revealed that the supplementation did not restore ichizinone production. This could be due to inefficient transport of the non-natural amino acids into the cells or disruptions in protein-protein interactions between the PKS and the NRPS assembly line.

The NRPS assembly line encoded by the genes *ichC*, *ichE*, *ichF* and *ichG* produces a linear tetrapeptide precursor, which undergoes further modifications to yield ichizinones. We propose that during this maturation process, the alpha carbon of beta-amino acid residue is oxidized, and the pyrazinone ring is formed through a cyclization reaction. The oxidation reaction might be catalyzed by the flavin-dependent oxidoreductase encoded by the gene *ichD*, while the cyclization may be mediated by the putative cyclase encoded by *ichJ*.

In this paper, we report the isolation of three new members of the pyrazinone family of natural products– ichizinones A (**9**), B (**10**), and C (**11**). The isolated ichizinones are tetrapeptides featuring a rare trisubstituted pyrazinone ring. The biosynthetic gene cluster responsible for the production of these compounds was identified through heterologous expression and gene inactivation studies. The structures of the isolated ichizinones, as well as the sequence of their biosynthetic gene cluster, indicate that the biosynthesis of tetrapeptide, trisubstituted pyrazinones substantially differs from the biosynthetic pathways of smaller dipeptide compounds. The results presented in this paper provide insights into the possible mechanisms involved in the production of ichizinones.

## Electronic supplementary material

Below is the link to the electronic supplementary material.

Supplementary Material 1

## Data Availability

No datasets were generated or analysed during the current study.
