# Supplementary material for: Identification and heterologous expression of an NRPS biosynthetic gene cluster responsible for the production of the pyrazinones Ichizinone A, B and C
Source: Microb Cell Fact. 2025 Jun 7;24:131. doi: 10.1186/s12934-025-02753-6 (PMC12144821; doi:10.1186/s12934-025-02753-6)
Supplement: Supplementary file 1 — Supplementary Material 1 [file 12934_2025_2753_MOESM1_ESM.docx]

**Supplementary Information**

**Identification and Heterologous Expression of a NRPS Bio-synthetic Gene Cluster Responsible for the Production of the Pyrazinones Ichizinone A, B and C**

Patrick Oberhäuser ^1, 2^, Maksym Myronovskyi ^1^, Marc Stierhof ^1^, Oleksandr Gromyko ^3,4^ and Andriy Luzhetskyy ^1,5^,*

1 – Department of Pharmaceutical Biotechnology, Saarland University, 66123 Saarbrücken; Germany, patrick.oberhaeuser@uni-saarland.de (P.O.); maksym.myronovskyi@uni-saarland.de (M.M.); marc.stierhof@uni-saarland.de (M.S.)

2 – INM – Leibniz Institute for New Materials, Campus D2 2, 66123 Saarbrücken, Germany, pat-rick.oberhaeuser@leibniz-inm.de

3 – Department of Genetics and Biotechnology, Ivan Franko National University of Lviv, 79005 Lviv, Ukraine

4 – Microbial Culture Collection of Antibiotic Producers, Ivan Franko National University of Lviv, 79005, oleksandr.gromyko@lnu.edu.ua

5 – Helmholtz Institute for Pharmaceutical Research Saarland, 66123 Saarbrücken, Germany

* – Correspondence: a.luzhetskyy@mx.uni-saarland.de; Tel.: +49-0681-70223

**Strains and Plasmids**

| **Table S1:** Bacterial strains used in this work | | |
| --- | --- | --- |
| **Strain** | **Description** | **Reference or source** |
| *Streptomyces* LV45-129 | The wild type strain; the source of the ichizinone cluster |  |
| *Streptomyces albus* Del14 | The heterologous host strain; cluster free derivative of the *S. albus* J1074 | [1] |
| *S. albus* E514 | Derivative of *S. albus* Del14 harboring the E514 cosmid | This work |
| *S. albus* E514_KOA | Derivative of *S. albus* Del14 harboring the E514_KOA cosmid | This work |
| *S. albus* E514_KOB | Derivative of *S. albus* Del14 harboring the E514_KOB cosmid | This work |
| *S. albus* E514_KOC | Derivative of *S. albus* Del14 harboring the E514_KOC cosmid | This work |
| *Escherichia coli* ET12567 pUB307 | Donor strain for intergeneric conjugation | [2] |
| *Escherichia coli* DH10β | General cloning strain | [3] |
|  |  |  |
|  | | |

| **Table S2:** Plasmids and cosmids used in this work | | |
| --- | --- | --- |
| **Strain** | **Description** | **Reference or source** |
| E514 | The cosmid containing 40 kb chromosomal fragment of *Streptomyces* LV45-129; contains ichizinone gene cluster | This work |
| E514_KOA | The derivative of E514 with the deletion of gene A | This work |
| E514_KOB | The derivative of E514 with the deletion of gene B | This work |
| E514_KOC | The derivative of E514 with the deletion of gene C | This work |
| pACS-hyg | The plasmid containing hygromycin resistance gene | [4] |
|  | | |

| **Table S3:** Primers used in this study | | |
| --- | --- | --- |
| **Primer** | **Sequence** |  |
| E514_KOA_F | ATGGCCTCCTCGCTGCTCGAAGCCGTAGACATCAAGGCGCCGGTCGCCGTGTTTAAACAATACTTGACATATCACTGT |  |
| E514_KOA_R | CTAGTCCCCGGTGCCTTCGGAGACCGTTCGTTCCGCCATCTTCTTGAATTGTTTAAACTCAGGCGCCGGGGGCGGTGT |  |
| E514_KOB_F | GTGGGCGACTGGATACGTTGCTGCCACCCCGCTCCCGACGCCGGGGTCCGGTTTAAACAATACTTGACATATCACTGT |  |
| E514_KOB_R | TCATCGCCTCCCGATCGGATCGGGAAGAGAGGAGGCGATGACGTCGGCGAGTTTAAACTCAGGCGCCGGGGGCGGTGT |  |
| E514_KOC_F | ATGACCGAGCCAGCACCCCGGGCCTCCGACGCCGACGCCACCCCCGAGCCGTTTAAACAATACTTGACATATCACTGT |  |
| E514_KOC_R | CTACTCCTCCGCCTCGAACTCCGCCAGGAGCGCGTCCAGCGAGTCCTCCGGTTTAAACTCAGGCGCCGGGGGCGGTGT |  |
| E514_KOA_chkF | TGGCAGCAACGTATCCAGTC |  |
| E514_KOA_chkR | GTCCGCCAGCTCCTCAGG |  |
| E514_KOB_chkF | GCTCGACGTTCTCCCACAG |  |
| E514_KOB_chkR | CGATCACCCTGTTCCTGGAG |  |
| E514_KOC_chkF | CACGCTGGTCGACCTGTTC |  |
| E514_KOC_chkR | CTTCTCCTGCCACGTCCTG |  |
|  | | |


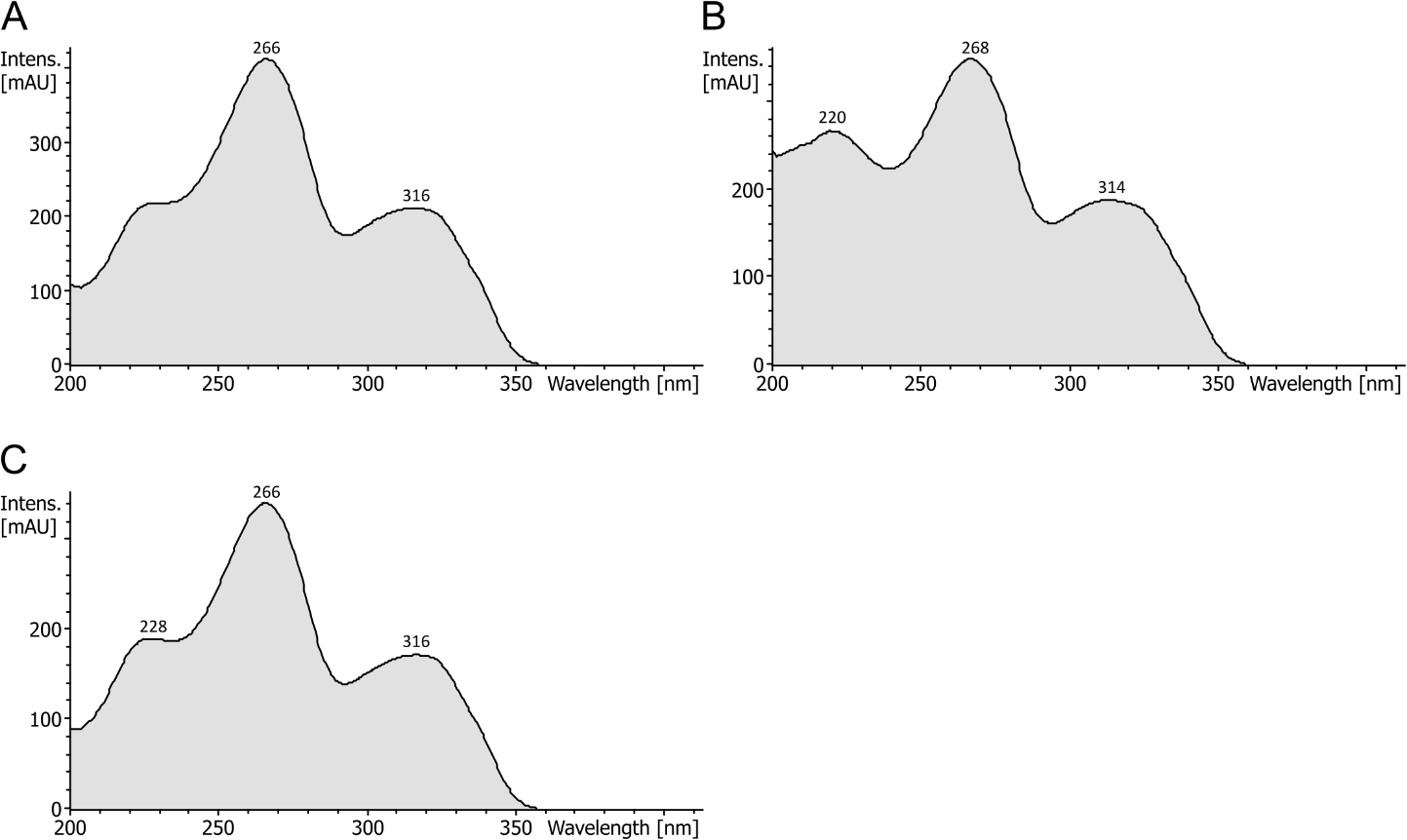


Figure S1: UV-Vis absorption spectra of isolated ichizinones. A – UV-Vis absorption spectrum of ichizinone A, B – UV-Vis absorption spectrum of ichizinone B, C – UV-Vis absorption spectrum of ichizinone C.

**NMR spectroscopy**


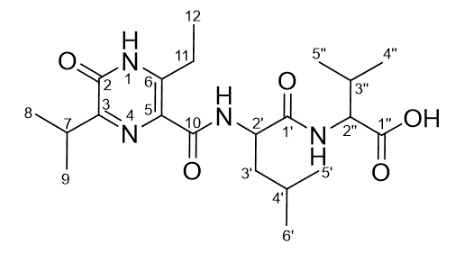
**^
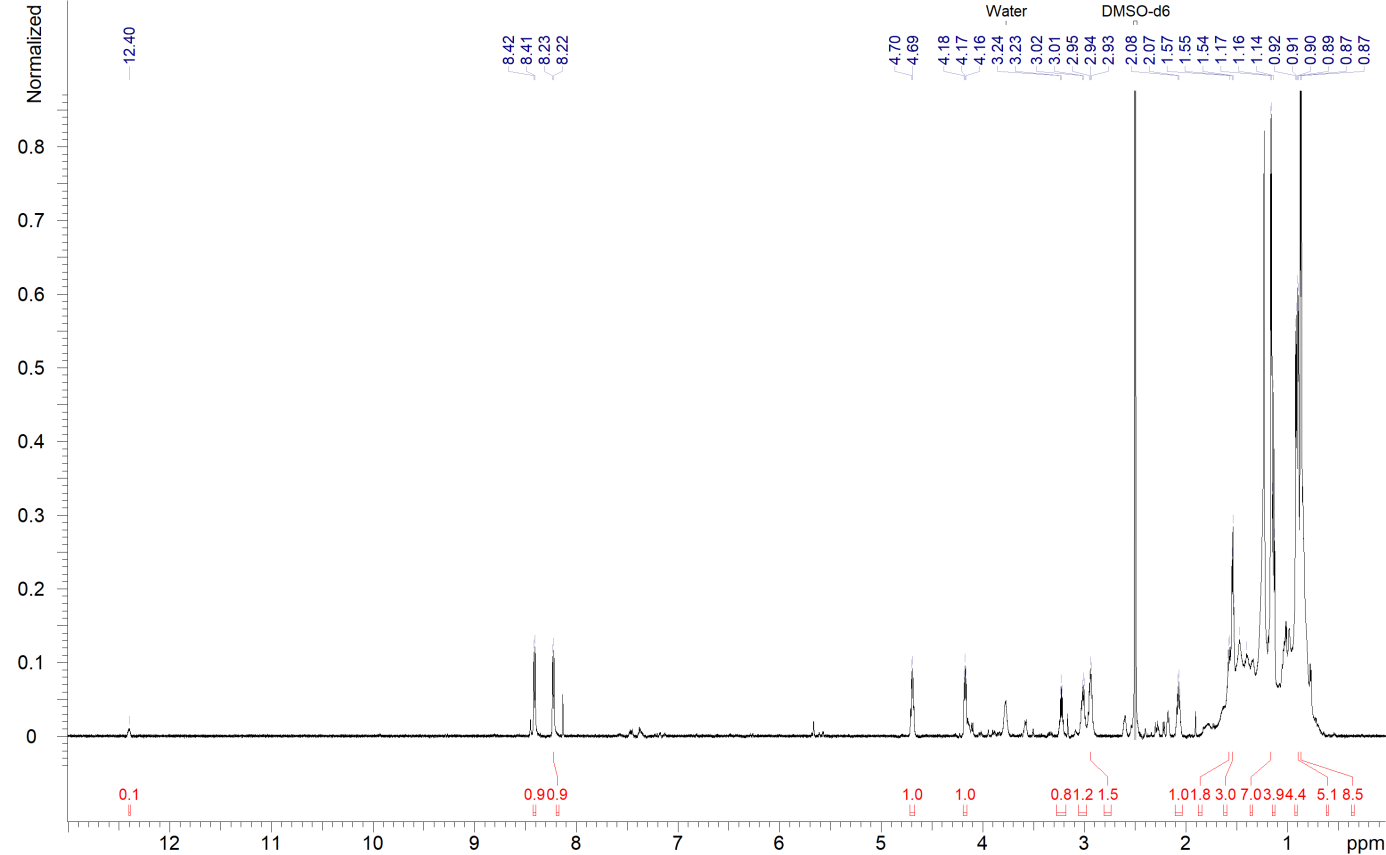
^**

Figure S2: ^1^H-NMR spectrum of ichizinone A in DMSO-d_6_ and TFA.

**^
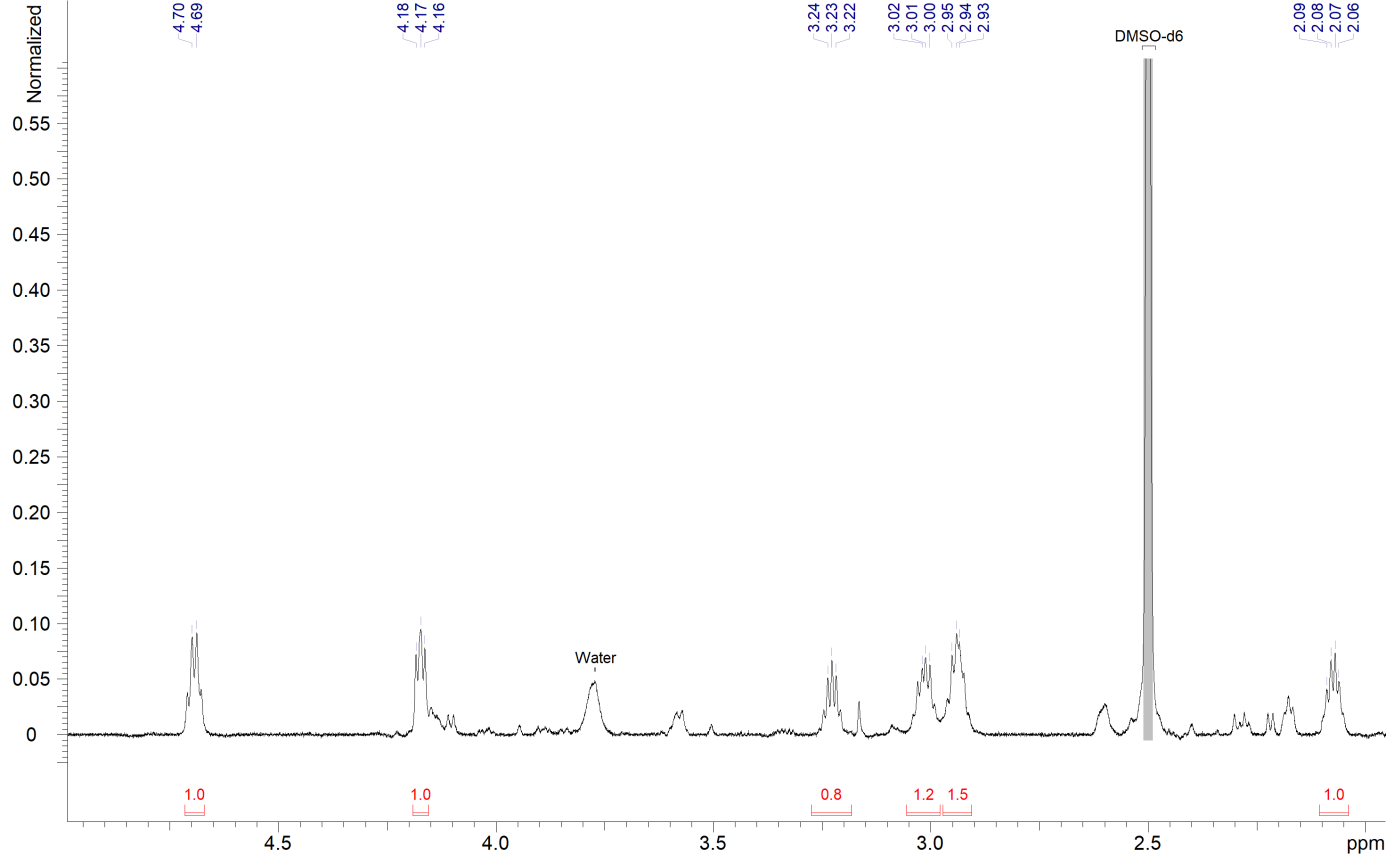
^**

Figure S3: ^1^H-NMR spectrum of ichizinone A in DMSO-d_6_ and TFA.


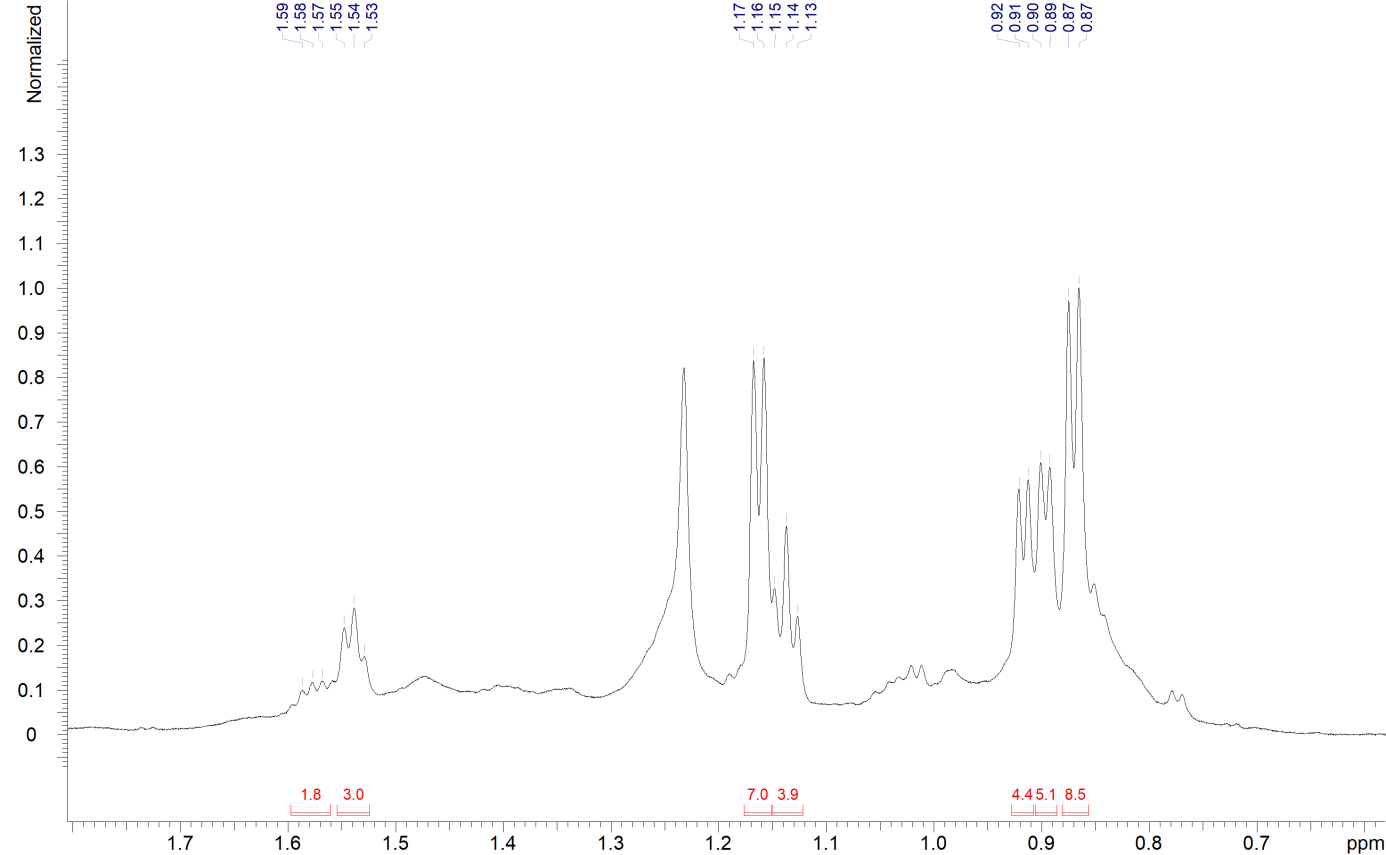


Figure S4: ^1^H-NMR spectrum of ichizinone A in DMSO-d_6_ and TFA.

**^
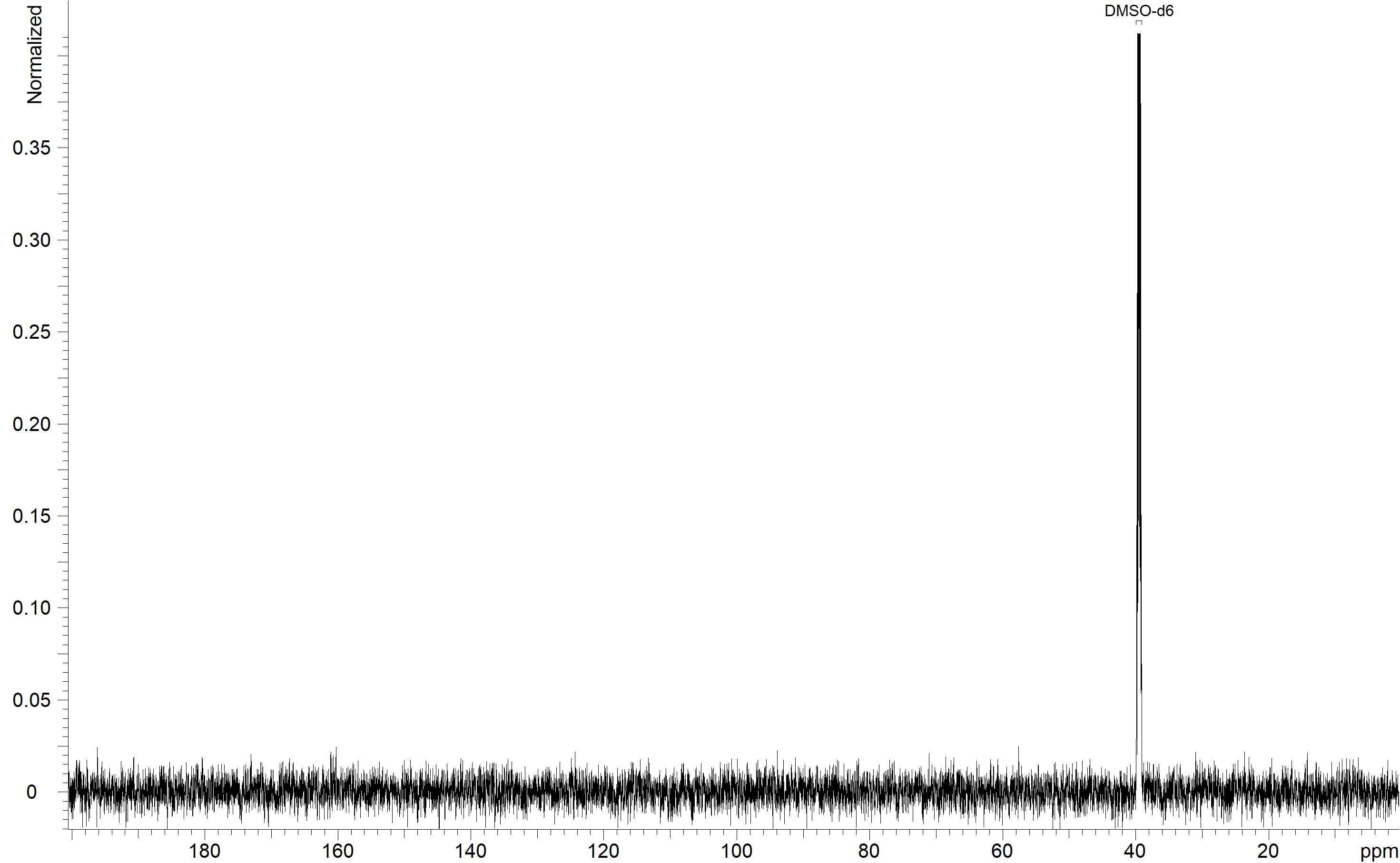
^**

Figure S5: ^13^C-NMR spectrum of ichizinone A in DMSO-d_6_ and TFA.


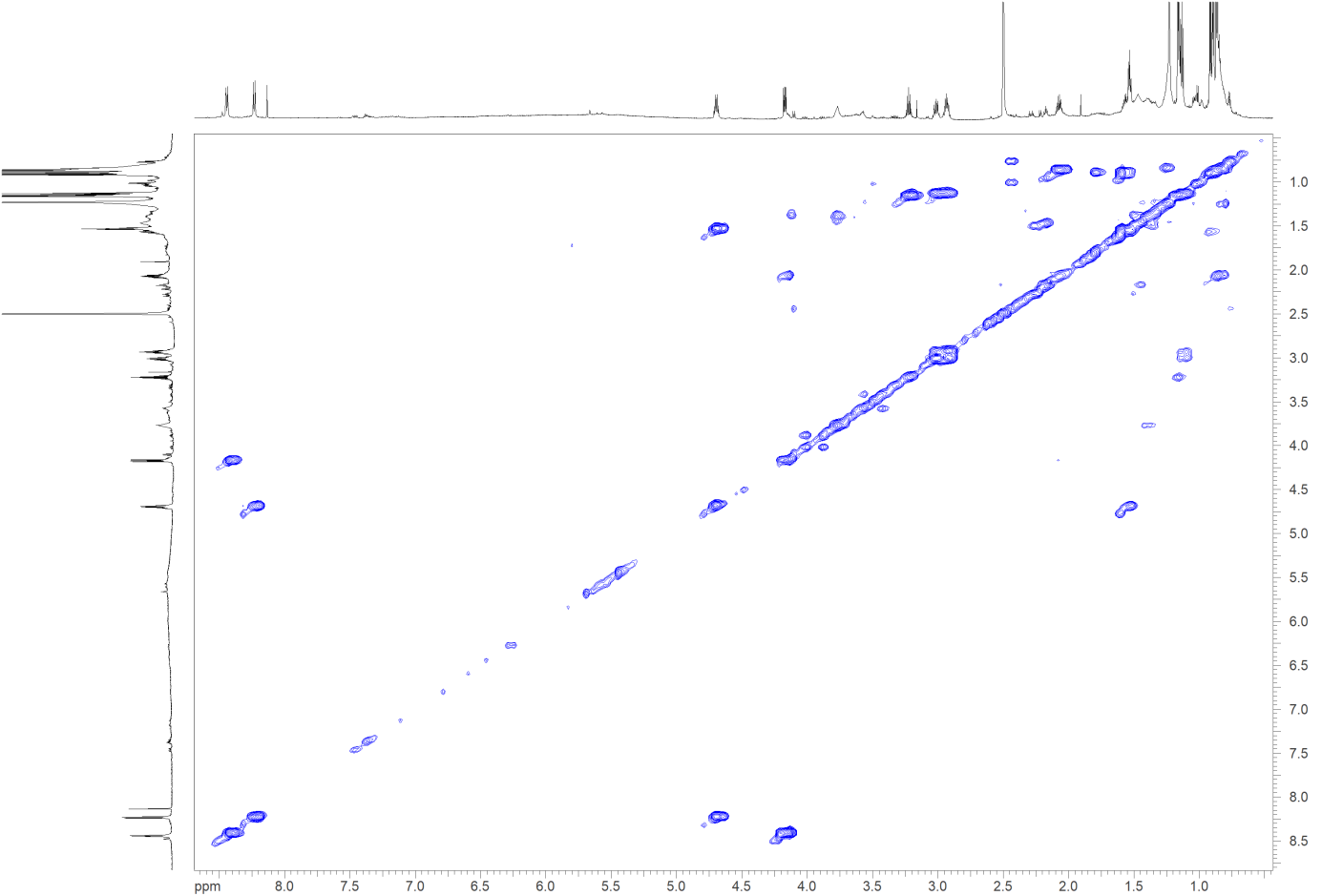


Figure S6: ^1^H-^1^H-COSY spectrum of ichizinone A in DMSO-d_6_ and TFA.


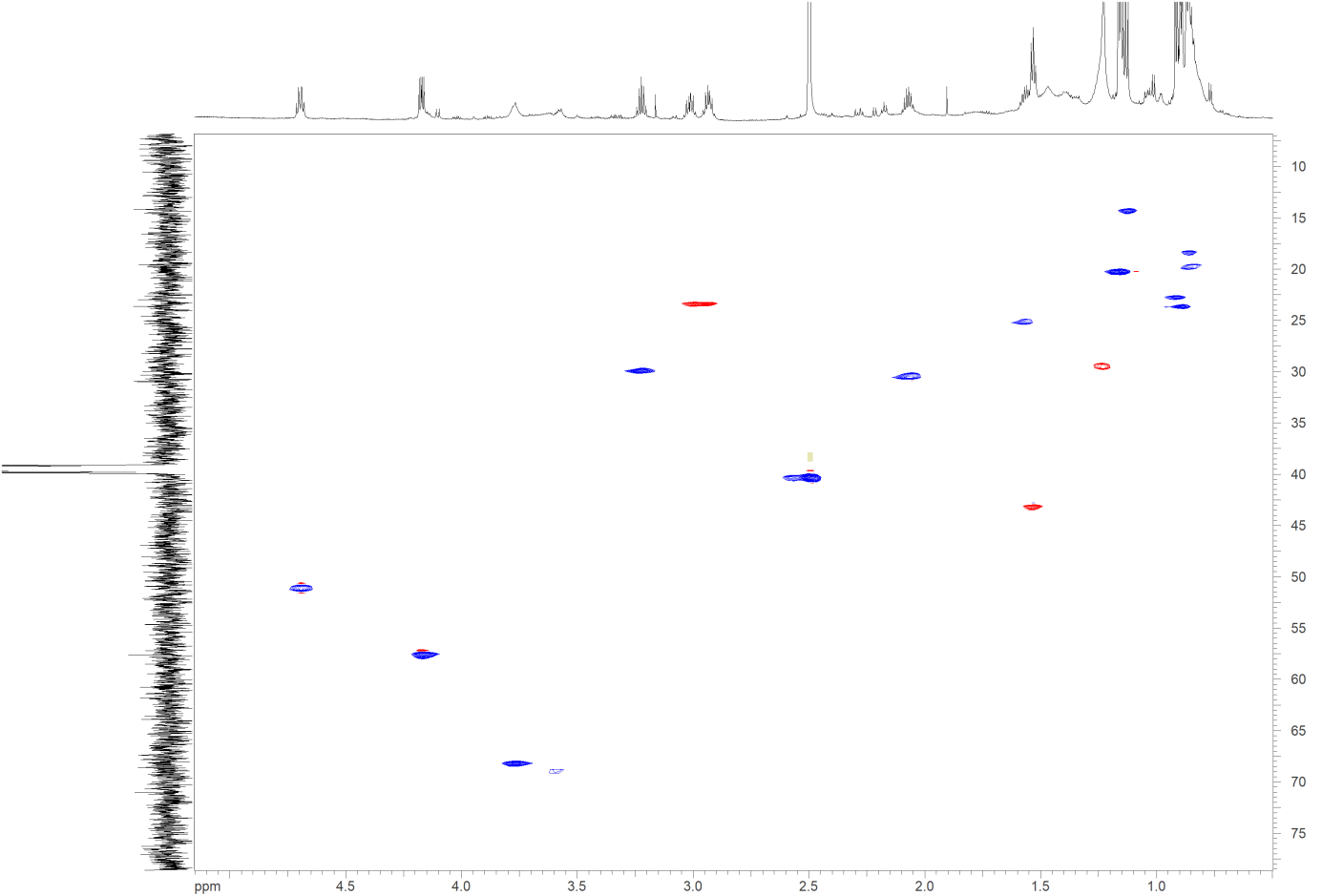


Figure S7: Edited HSQC spectrum of ichizinone A in DMSO-d_6_ and TFA .


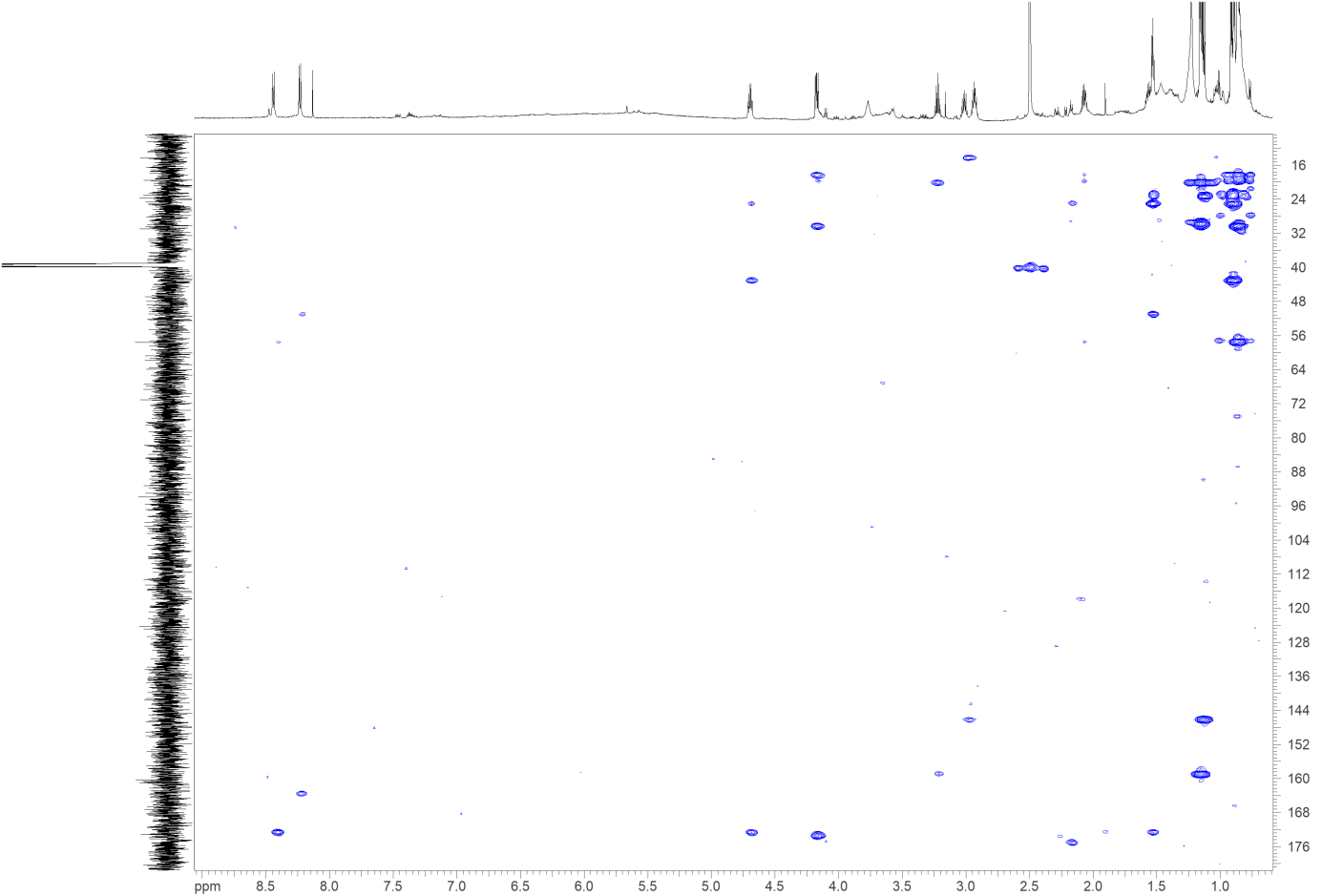


Figure S8: HMBC spectrum of ichizinone A in DMSO-d_6_ and TFA.

*
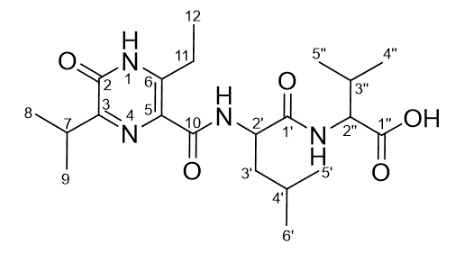
*
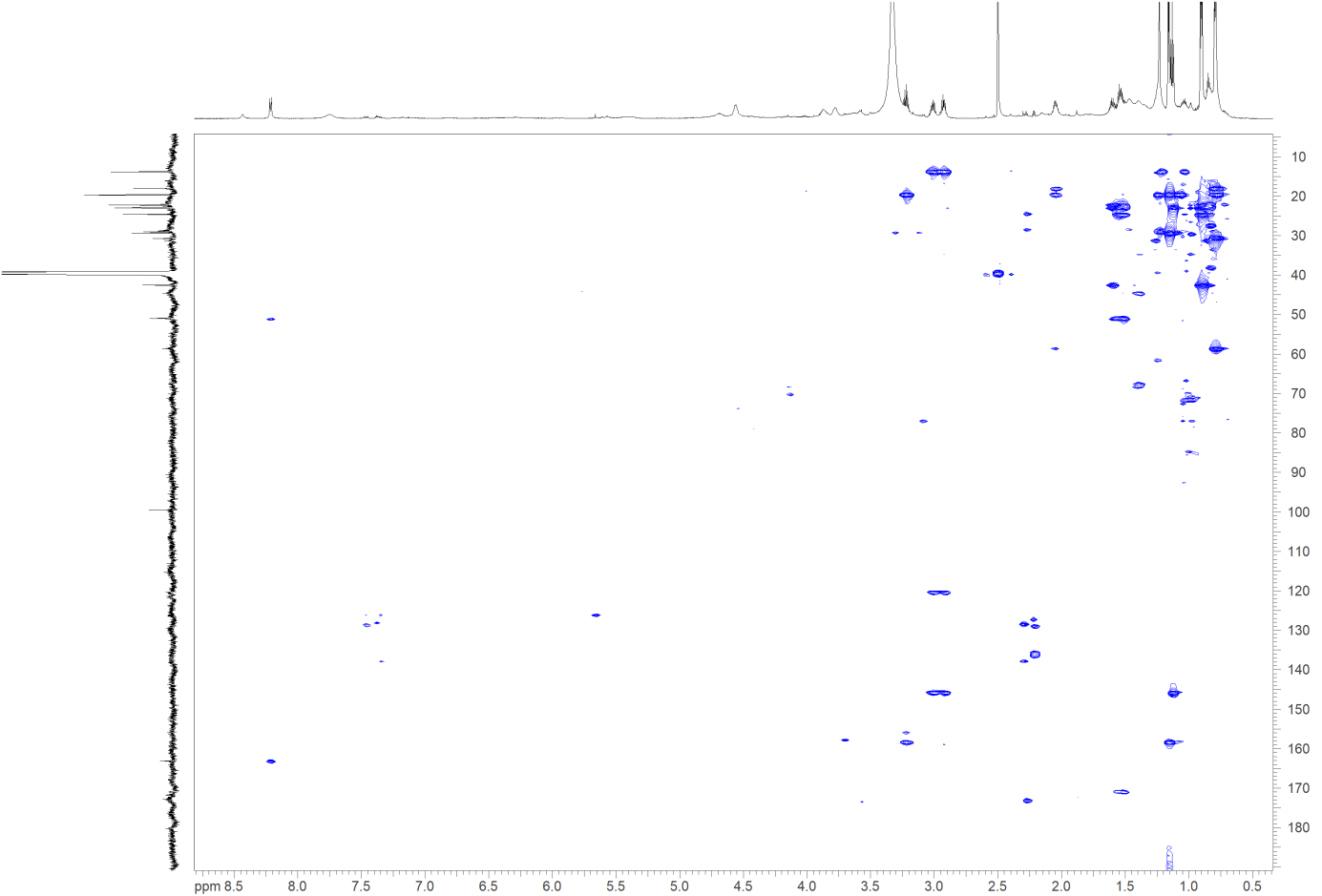


7 🡪 2

11 🡪 5

Figure S9: HMBC spectrum of key correlations of ichizinone A in DMSO-d_6_; HMBC correlation H7 🡪 C2 and H11 🡪 C5 were not observed when TFA was added.

**Table S4**: NMR Table of ichizinone B measured in DMSO-d_6._

| **Ichizinone B** | | | | |
| --- | --- | --- | --- | --- |
| **No, type** | **δ(^13^C, ^15^N) [ppm]** | **δ(^1^H) [ppm], mult (J)** | **COSY   (H-)** | **HMBC  (C-/N-)** |
| 1-NH |  |  |  |  |
| 2-C | n.a.^*^ |  |  | n.a.^*^ |
| 3-C | 158.9 |  |  | 7, 8, 9 |
| 4-N |  |  |  |  |
| 5-C | 120.8 |  |  | 11 |
| 6-C | 142.5 |  |  | 11 |
| 7-CH | 29.4 | 3.22, dt (13.7, 6.9) | 8, 9 | 8, 9 |
| 8-CH_3_ | 19.7 | 1.16, d (6.8) | 7 | 7, 9 |
| 9-CH_3_ | 19.6 | 1.15, d (6.8) | 7 | 7, 8 |
| 10-C | 163.4 | - |  | 2’-NH |
| 11-CH_2_ | 34.2 | 4.42, q (13.5) |  | 13/17 |
| 12-C | 138.2 | - |  | 12, 14, 16 |
| 13/17-CH | 128.7 | 7.34, d (7.5) | 14/16 | 11, 14/16, 15 |
| 14/16-CH | 128.3 | 7.25, t (7.5) | 13/17, 15 | 13/17, 15 |
| 15-CH | 126.4 | 7.20, t (7.5) | 14/16 | 13/17, 14/16 |
| **Leu** |  |  |  |  |
| 1’-C | 171.0 |  |  | 2’-NH , 2', 3' |
| 2’-CH | 51.0 | 4.62, m | 2’-NH, 3' | 2’-NH , 3', 4' |
| 3’-CH_2_ | 42.7 | 1.54, m | 2', 4' | 2', 4', 5', 6' |
| 4’-CH | 24.6 | 1.59, m | 3', 5', 6' | 3', 5', 6' |
| 5’-CH_3_ | 22.2 | 0.82, d (6.0) | 4' | 3', 4', 6' |
| 6’-CH_3_ | 23.0 | 0.89, d (5.8) | 4' | 3', 4', 5' |
| 2’-NH |  | 8.30, d (8.6) | 2' | - |
| **Val** |  |  |  |  |
| 1’’-C | 172.4 |  |  | 3'' |
| 2’’-CH | 58.7 | 3.90, ovl.^**^ |  | 4'', 5'' |
| 3’’-CH | 30.6 | 2.05, m | 4'', 5'' | 4'', 5'' |
| 4’’-CH_3_ | 19.6 | 0.80, d (6.9) | 3'' | 3'', 5'' |
| 5’’-CH_3_ | 18.1 | 0.80, d (6.9) | 3'' | 3'', 4'' |
| 2’’-NH |  |  |  |  |
| ^*^n.a. = no signal or correlation available ^**^ovl. = overlap with other signals | | | | |


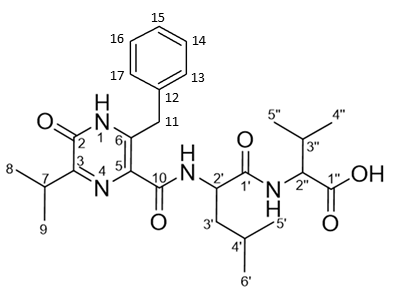


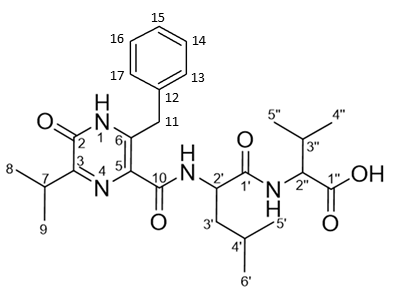

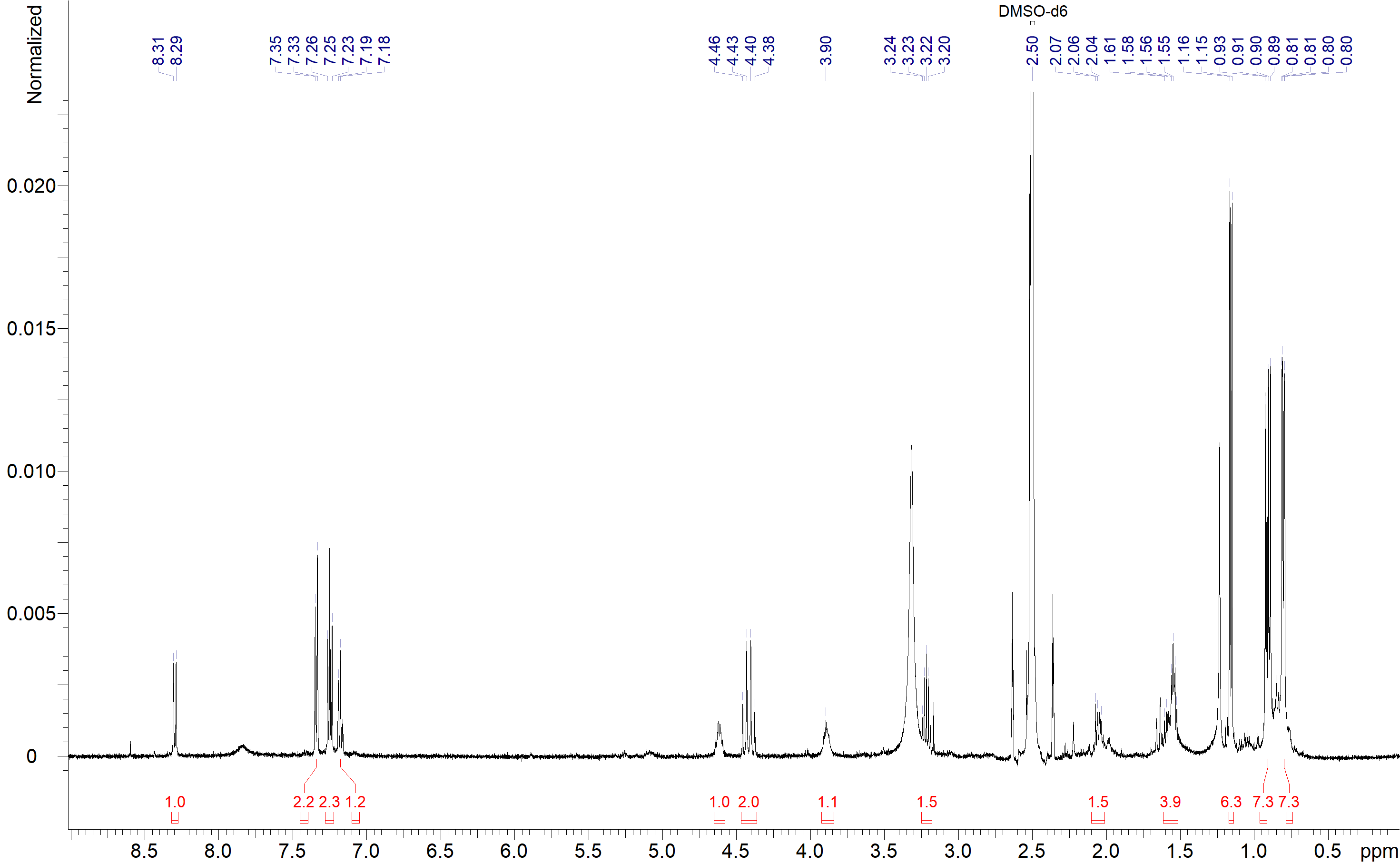


Figure S10: ^1^H-NMR spectrum of ichizinone B in DMSO-d_6_.


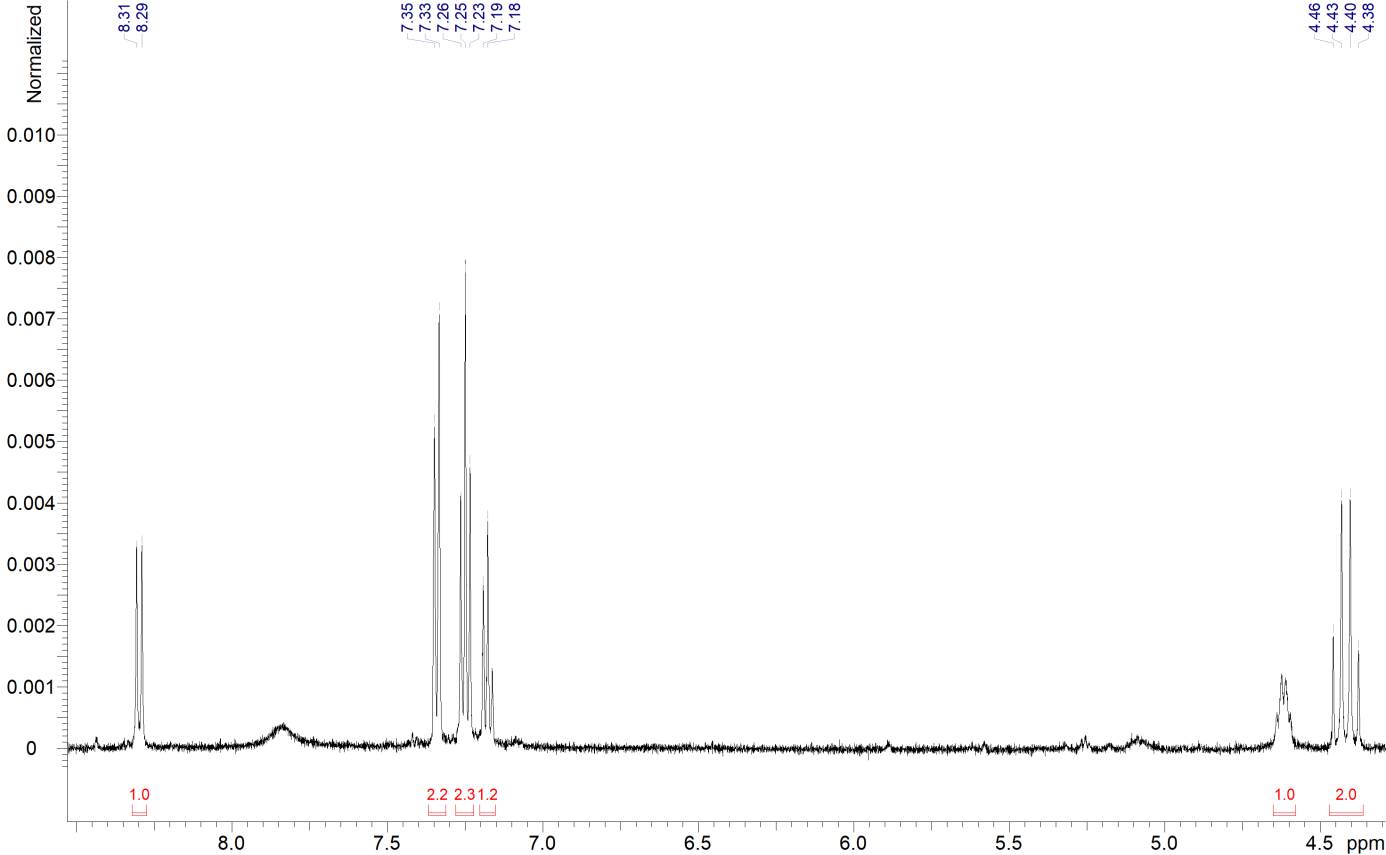


Figure S11: ^1^H-NMR spectrum of ichizinone B in DMSO-d_6_.


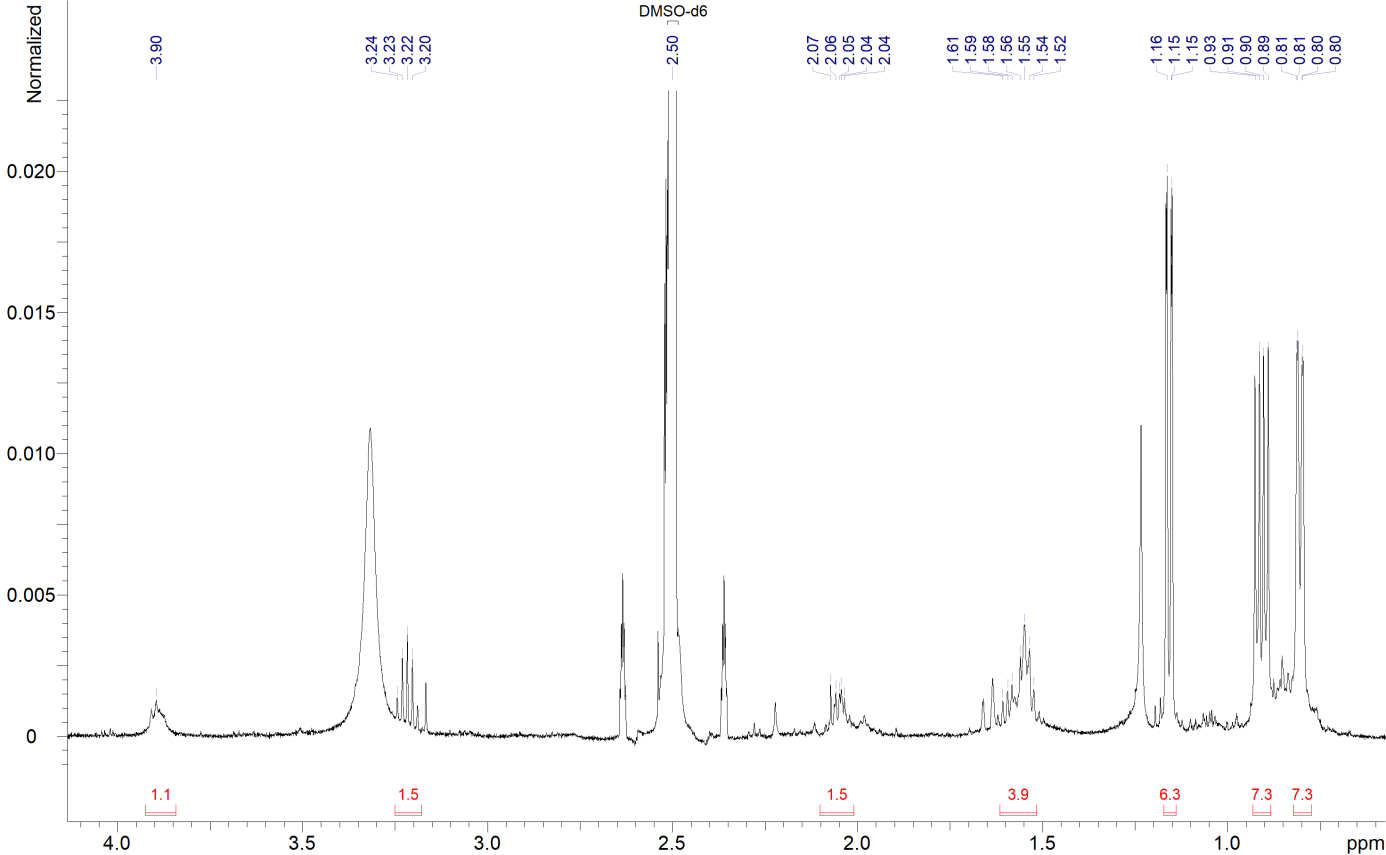


Figure S12: ^1^H-NMR spectrum of ichizinone B in DMSO-d_6_.


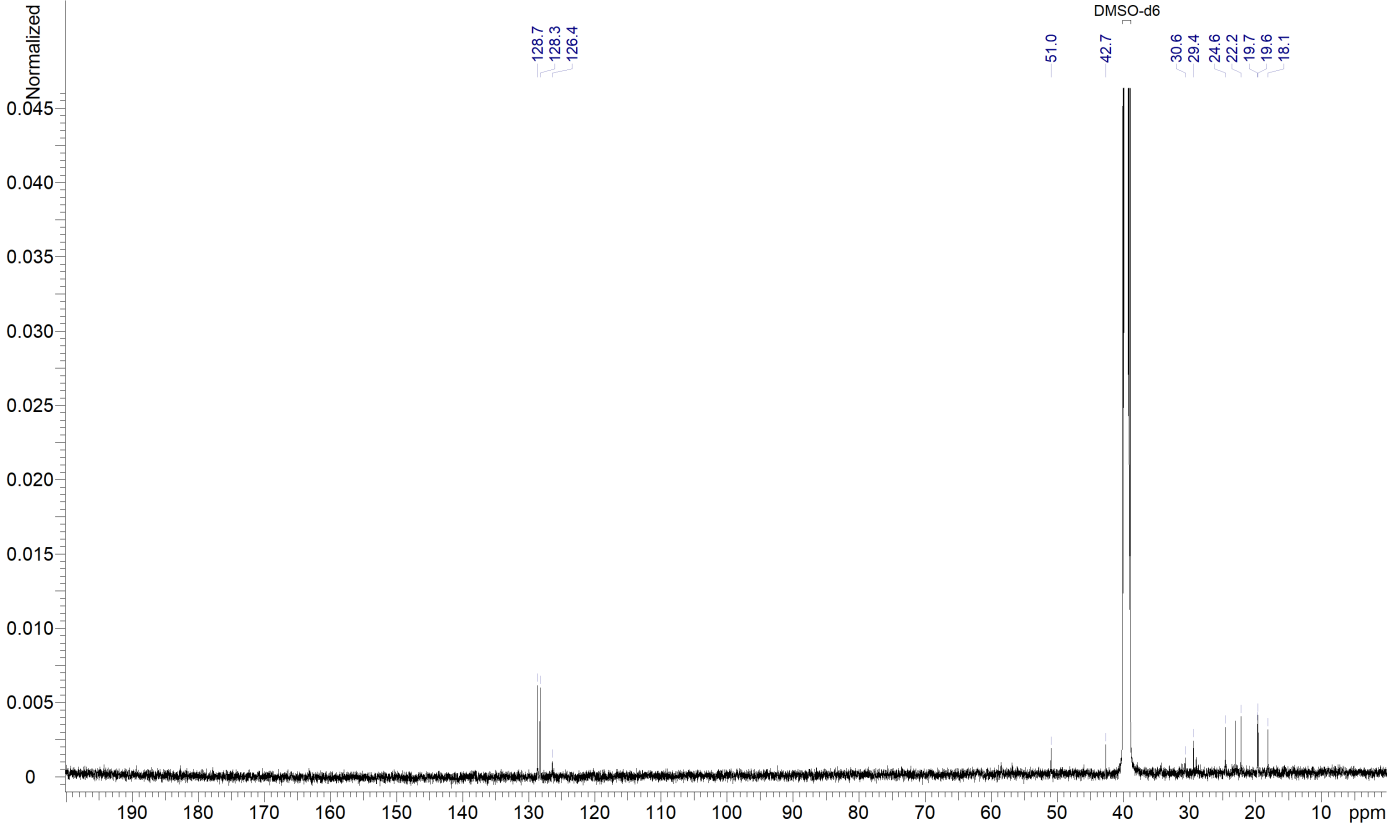


Figure S13: ^13^C-NMR spectrum of ichizinone B in DMSO-d_6_.


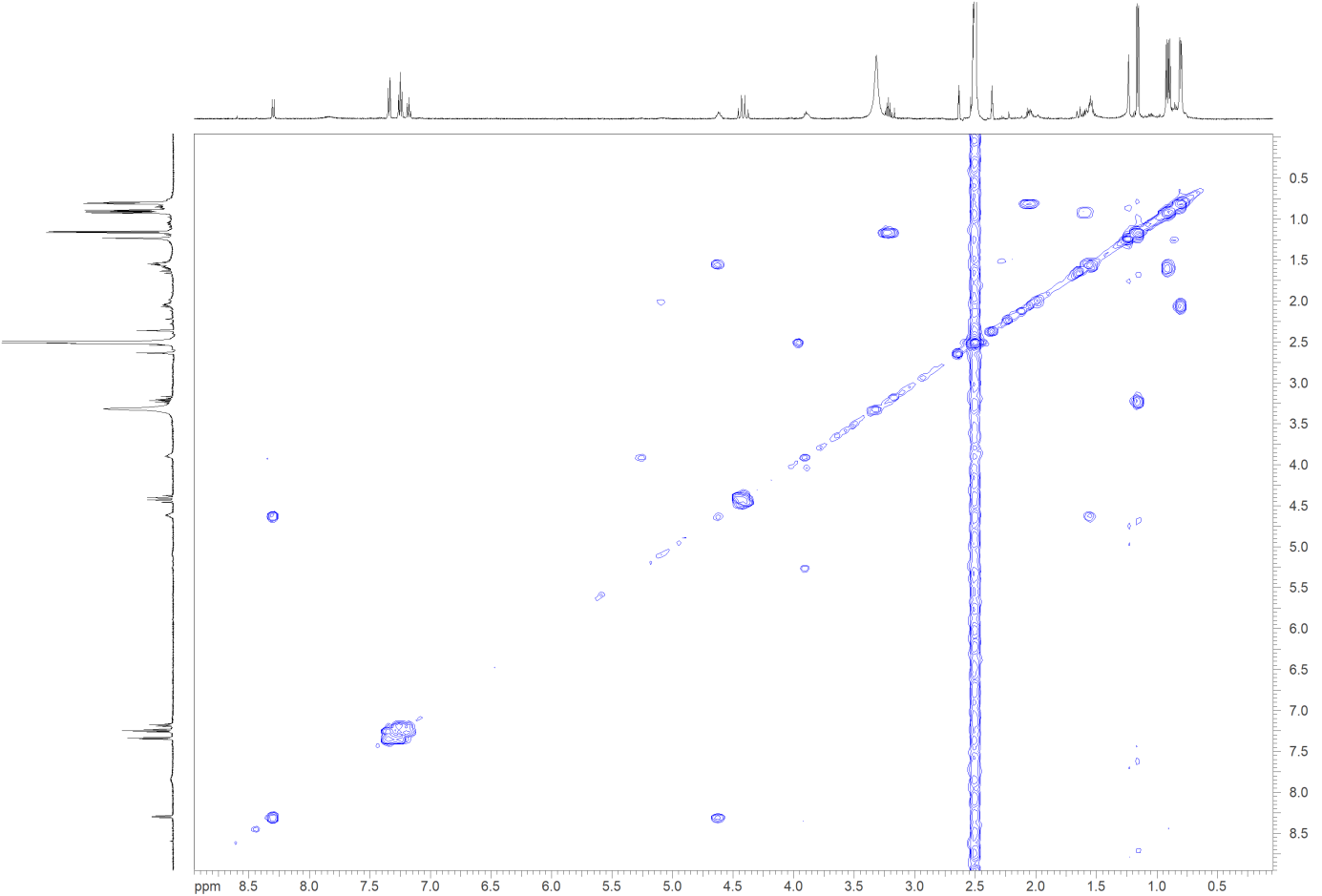


Figure S14: ^1^H-^1^H-COSY spectrum of ichizinone B in DMSO-d_6_.


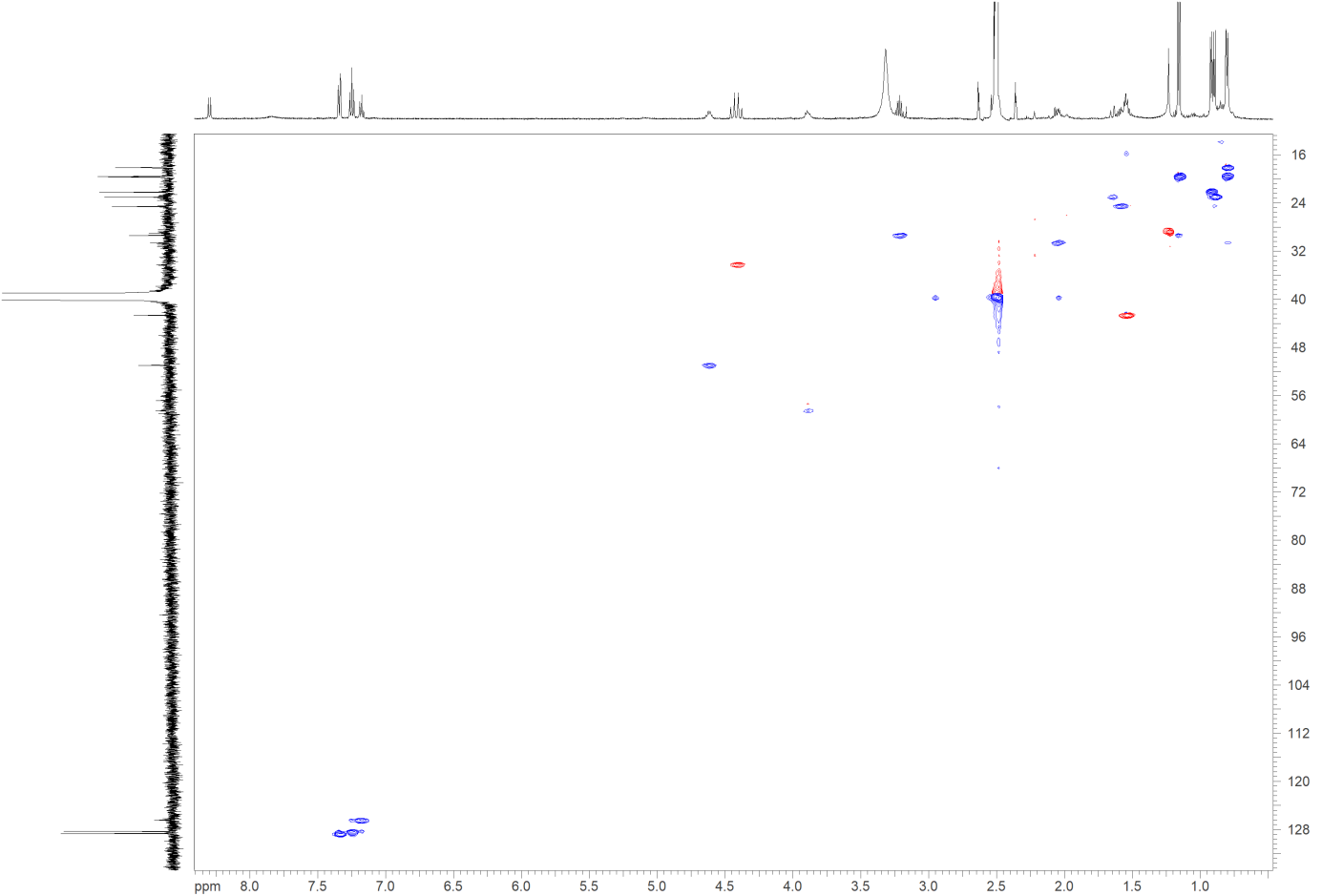


Figure S15: Edited HSQC spectrum of ichizinone B in DMSO-d_6_.


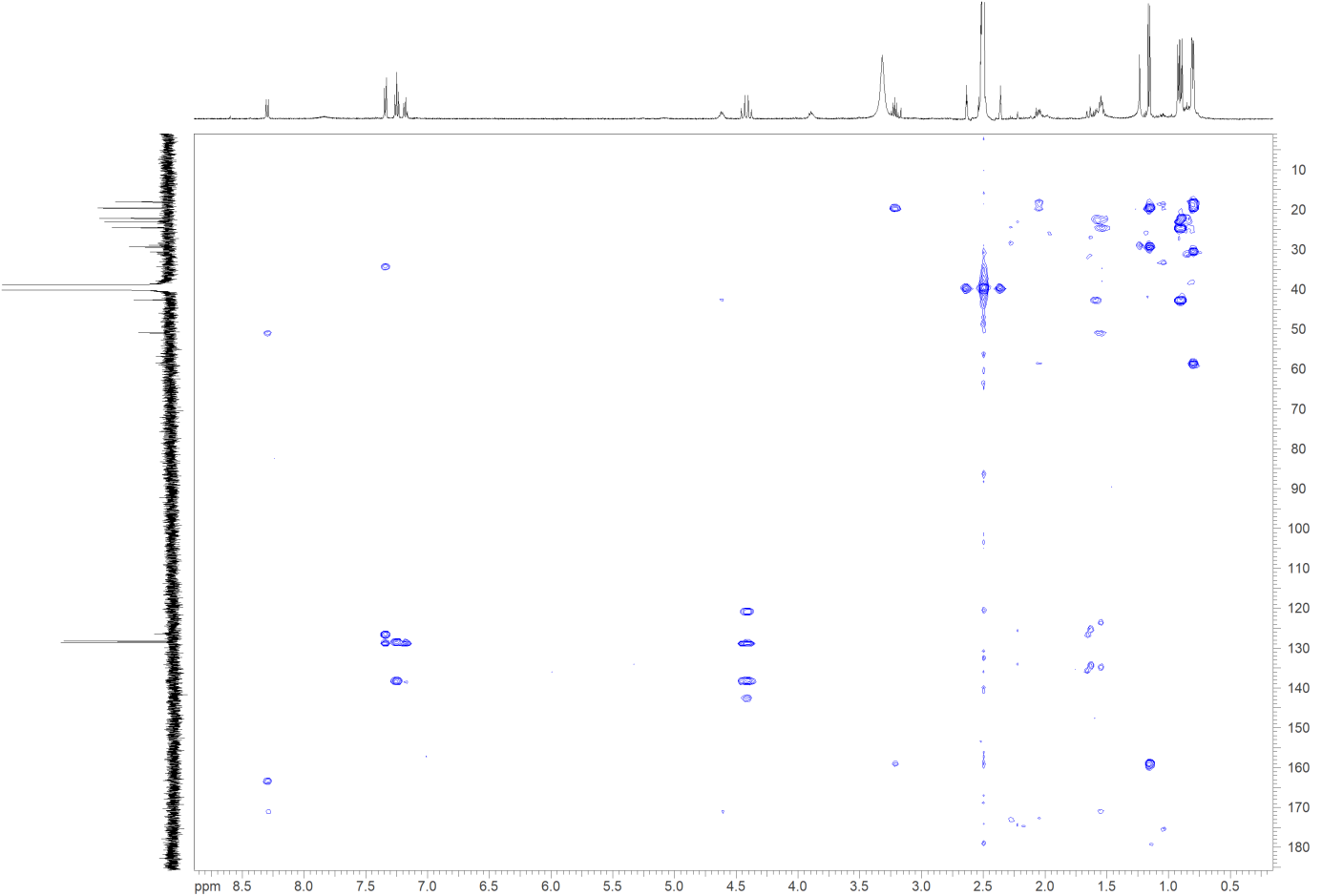


Figure S16: HMBC spectrum of ichizinone B in DMSO-d_6_.


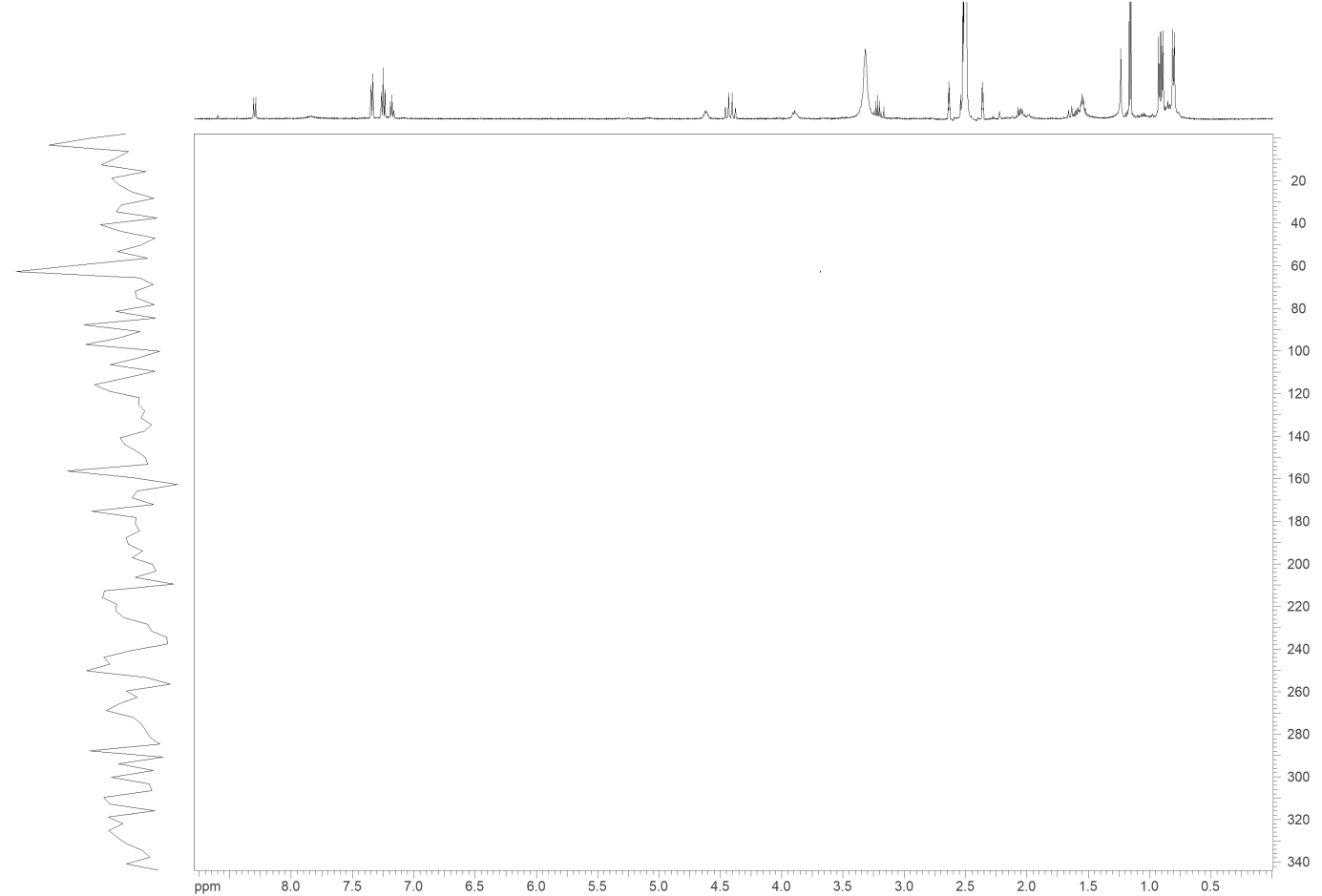


Figure S17: ^15^N-HMBC spectrum of ichizinone B in DMSO-d_6_.

**Table S5**: NMR Table of ichizinone C measured in MeOD-d_3_ and JBIR-57 for comparison.

| **Ichizinone C** | | | | | **JBIR-57** | |
| --- | --- | --- | --- | --- | --- | --- |
| **No, type** | **δ(^13^C, ^15^N) [ppm]** | **δ(^1^H) [ppm], mult(J)** | **COSY   (H-)** | **HMBC  (C-/N-)** | **δ(^13^C, ^15^N) [ppm]** | **δ(^1^H) [ppm], mult(J)** |
| 1-NH | 179.65 |  |  | 11 |  |  |
| 2-C | 157.93 |  |  | 7 | 155.8 |  |
| 3-C | 160.41 |  |  | 7, 8, 9 | 158.3 |  |
| 4-N | 320.15 |  |  | 7, 2’-NH, 11 |  |  |
| 5-C | 123.37 |  |  | 2’-NH, 11 | 120.9 |  |
| 6-C | 142.24 |  |  | 11 | 140.8 |  |
| 7-CH | 31.17 | 3.32, m | 8, 9 | 8, 9 | 29.5 | 3.21, q (6.8) |
| 8-CH_3_ | 20.20 | 1.24, d (6.9) | 7 | 7, 9 | 19.9 | 1.15, d (6.8) |
| 9-CH_3_ | 20.25 | 1.25, d (6.9) | 7 | 7, 8 | 19.8 | 1.15, d (6.8) |
| 10-C | 166.43 |  |  | 2’-NH, 2' | 163.6 |  |
| 11-CH_3_ | 17.12 | 2.61, s |  |  | 16.7 | 2.53, s |
| **Leu** |  |  |  |  | **Leu** |  |
| 1’-C | 174.92 |  |  | 2’-NH, 2’’-NH, 2', 3' | 171.6 |  |
| 2’-CH | 53.14 | 4.66, dt (7.6, 6.8) | 2’-NH, 3' | 2’-NH, 3' ,4' | 50.6 | 4.53, dd (14.8, 8.4) |
| 3’-CH_2_ | 43.06 | 1.70, ovl^*^ | 2', 4' | 2’-NH, 2', 4', 5', 6' | 42.6 | 1.52, dd (14.8, 6.2) |
| 4’-CH | 26.35 | 1.71, ovl^*^ | 3', 5', 6' | 2', 3', 5', 6' | 24.7 | 1.56, q (6.2) |
| 5’-CH_3_ | 22.51 | 0.98, d (6.2) | 4' | 3', 4', 6' | 23.3 | 0.89, d (6.2) |
| 6’-CH_3_ | 23.52 | 0.99, d (6.4) | 4' | 3', 4', 5' | 22.4 | 0.88, d (6.2) |
| 2’-NH | 117.54 | 8.34, d (8.2) | 2' | 2', 3', 4' |  | 8.16, d (8.4) |
| **Val** |  |  |  |  | **Ala** |  |
| 1’’-C | 174.85 |  |  | 2'', 3'' | 174.1 |  |
| 2’’-CH | 59.29 | 4.33, dd (8.7, 5.7) | 2’’-NH, 3'' | 2’’-NH, 3'', 4'', 5'' | 47.8 | 4.14, dq (7.0, 6.2) |
| 3’’-CH | 31.86 | 2.19, m | 2'', 4'', 5'' | 2’’-NH, 2'', 4'', 5'' | 17.7 | 1.24, d (7.0) |
| 4’’-CH_3_ | 18.37 | 0.95, d (6.8) | 3'' | 2'', 3'', 5'' |  |  |
| 5’’-CH_3_ | 19.73 | 0.97, d (7.0) | 3'' | 2'', 3'', 4'' |  |  |
| 2’’-NH | 117.54 | 8.16, d (8.5) | 2'' | 2'', 3'' |  | 8.41, br s |


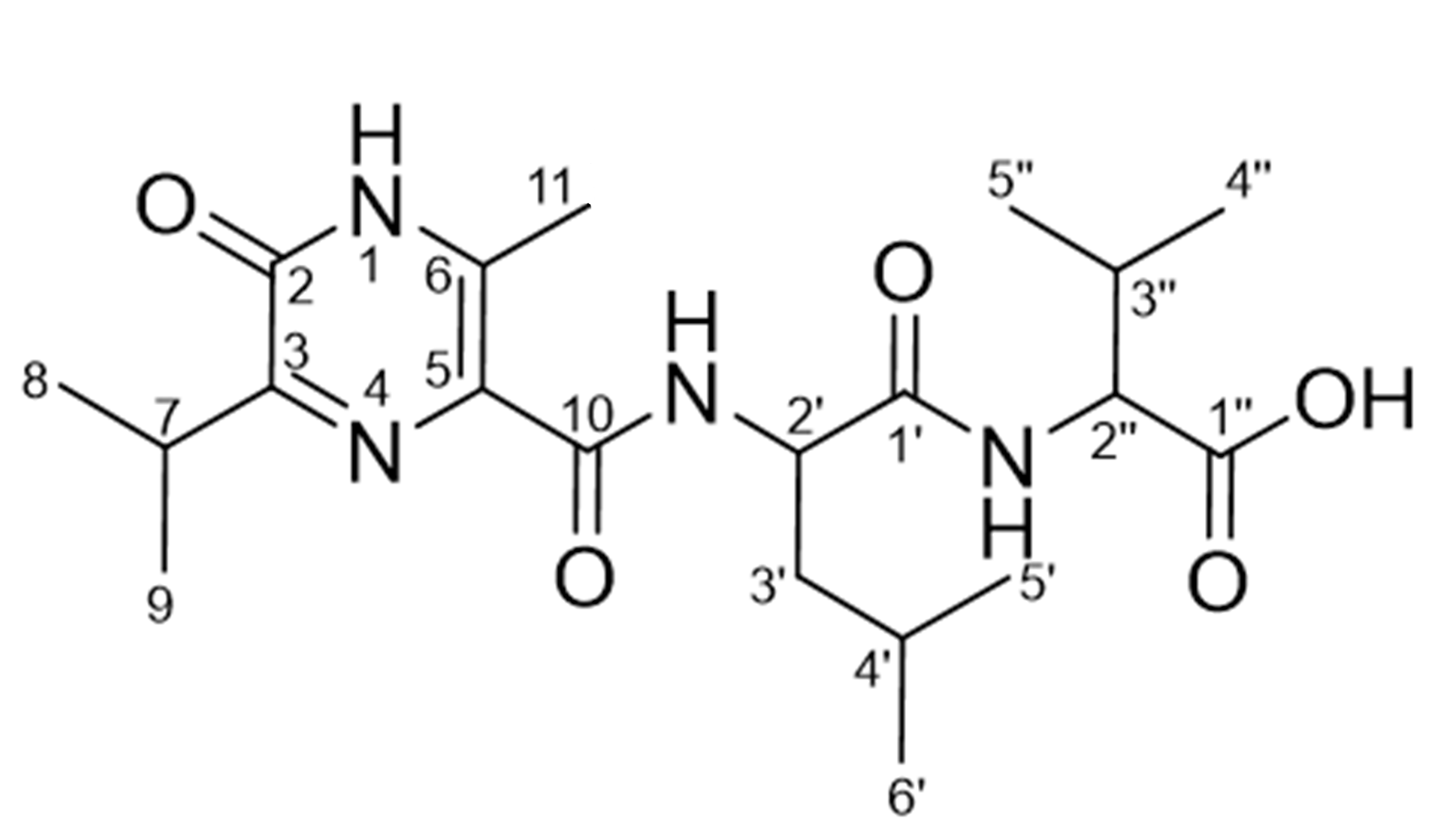


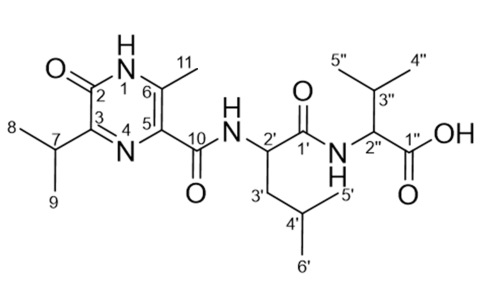

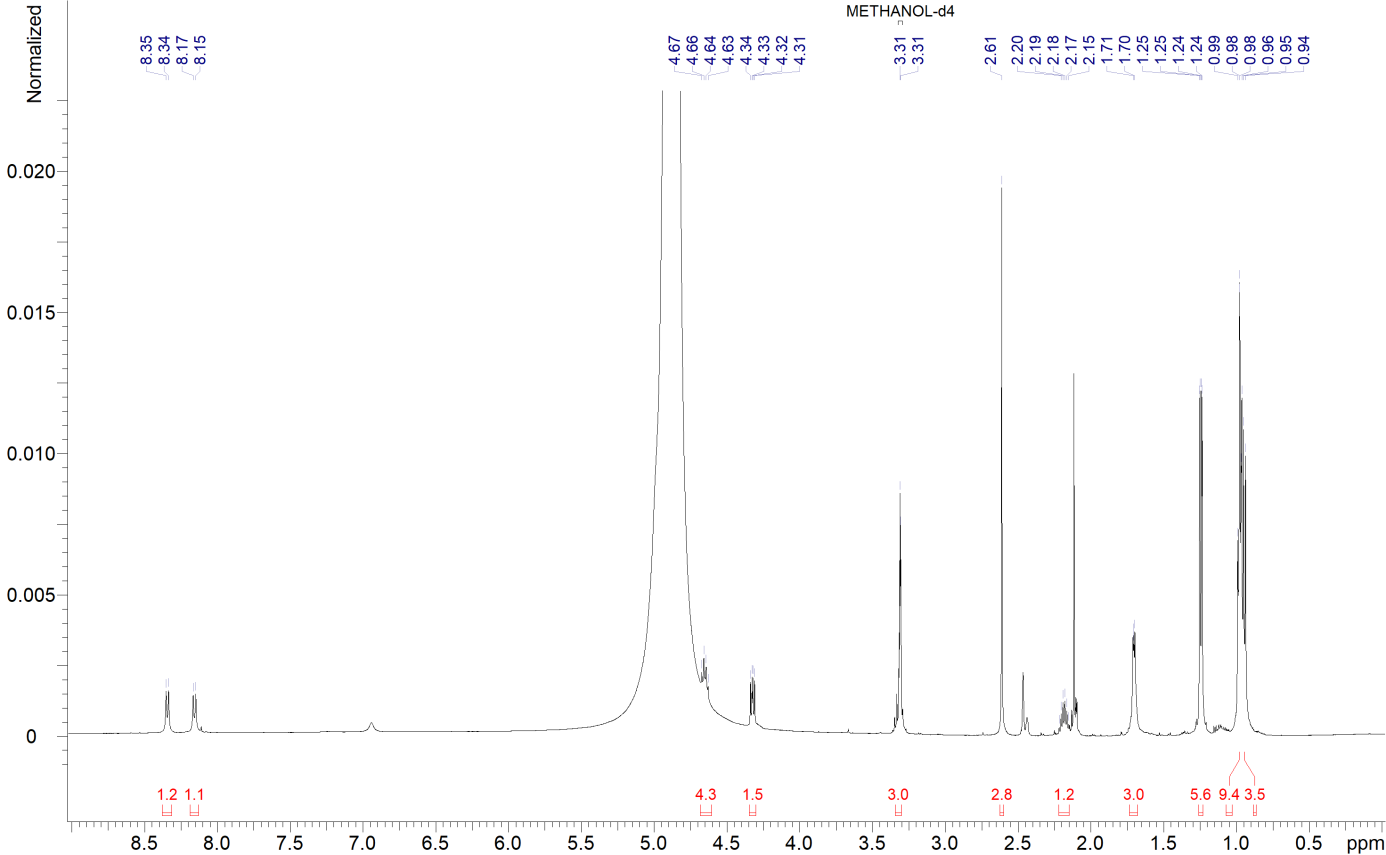


Figure S18: ^1^H-NMR spectrum of ichizinone C in MeOD-d_3_.


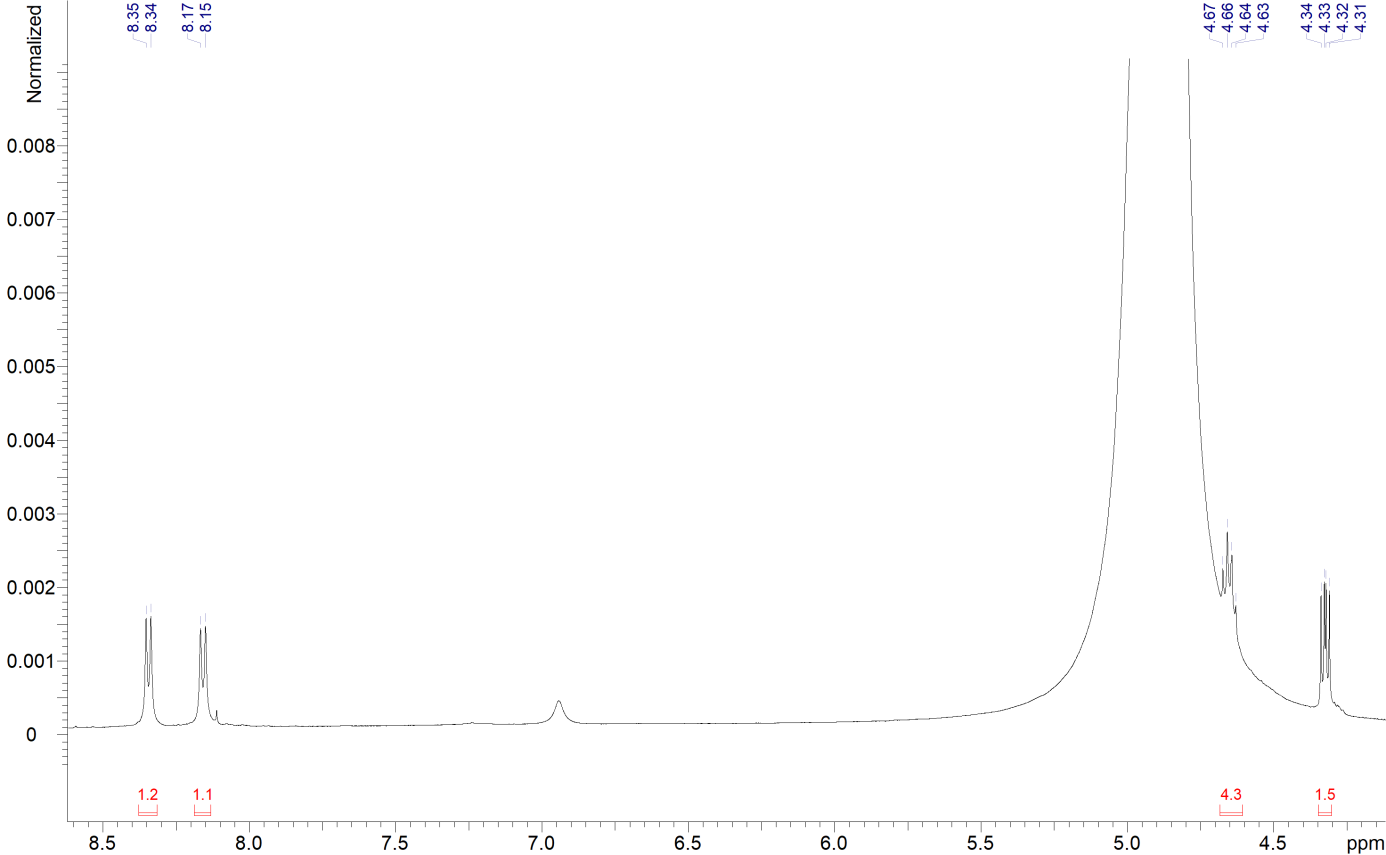


Figure S19: ^1^H-NMR spectrum of ichizinone C in MeOD-d_3_.


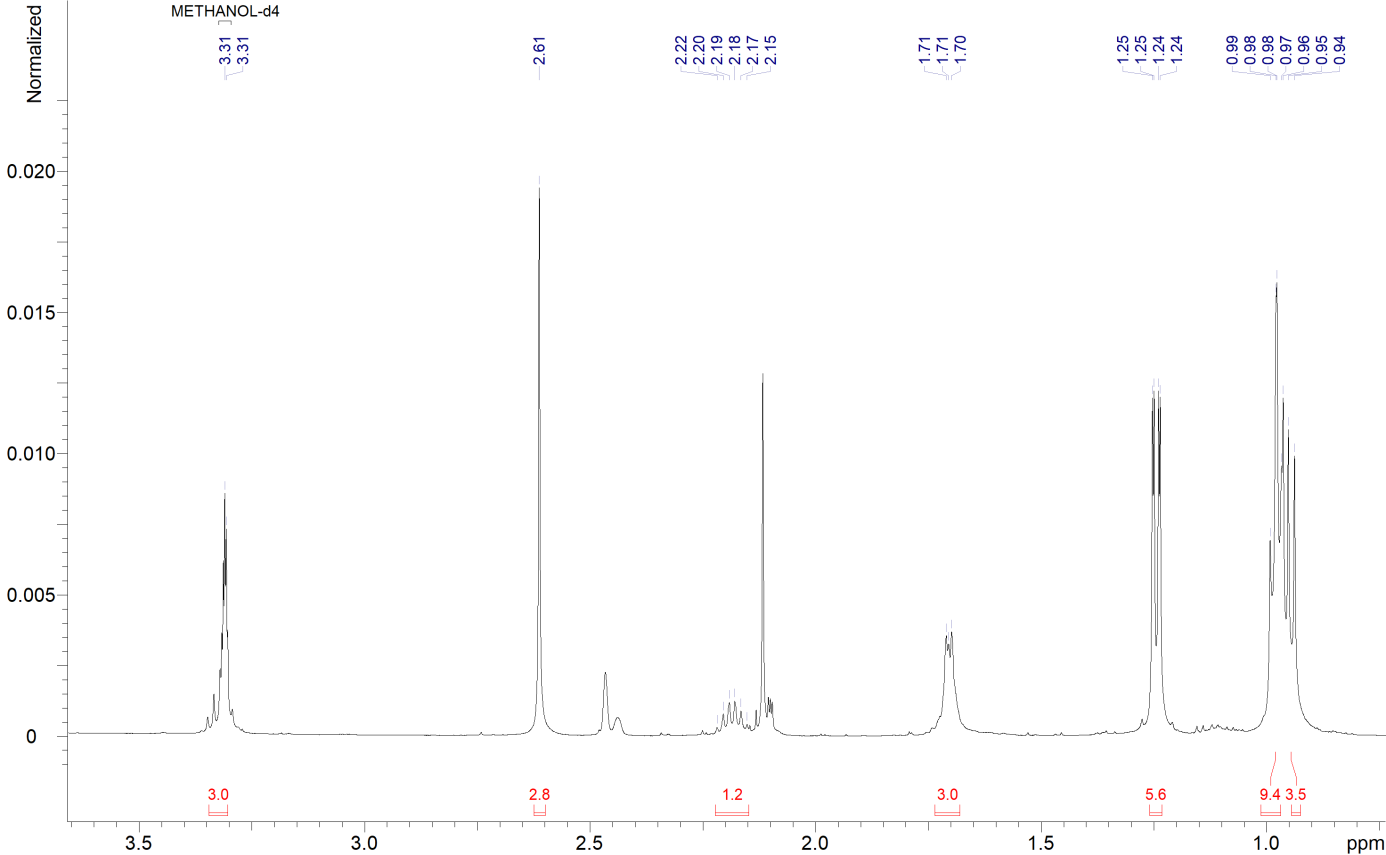


Figure S20: ^1^H-NMR spectrum of ichizinone C in MeOD-d_3_.


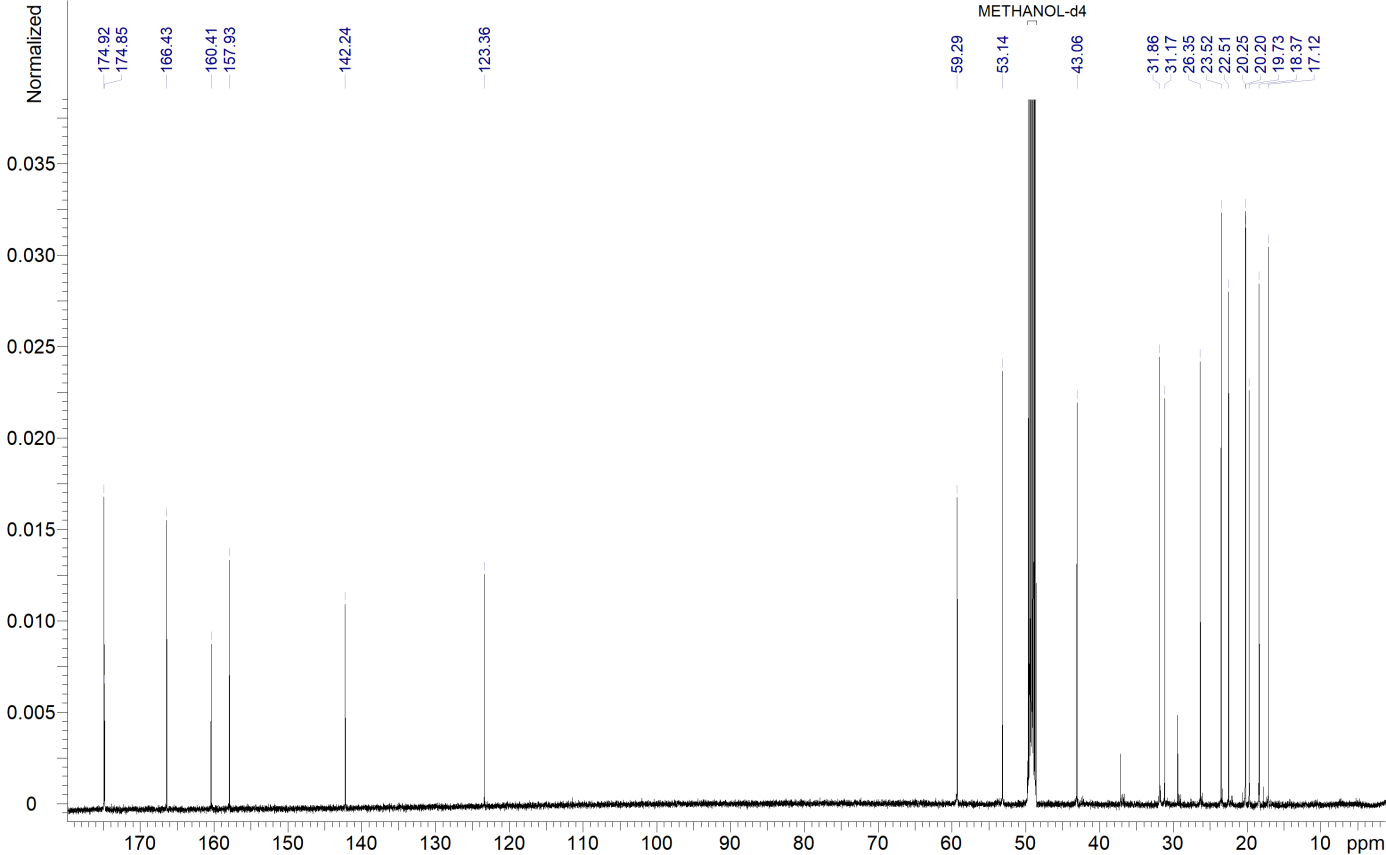


Figure S21: ^13^C-MR spectrum of ichizinone C in MeOD-d_3_.


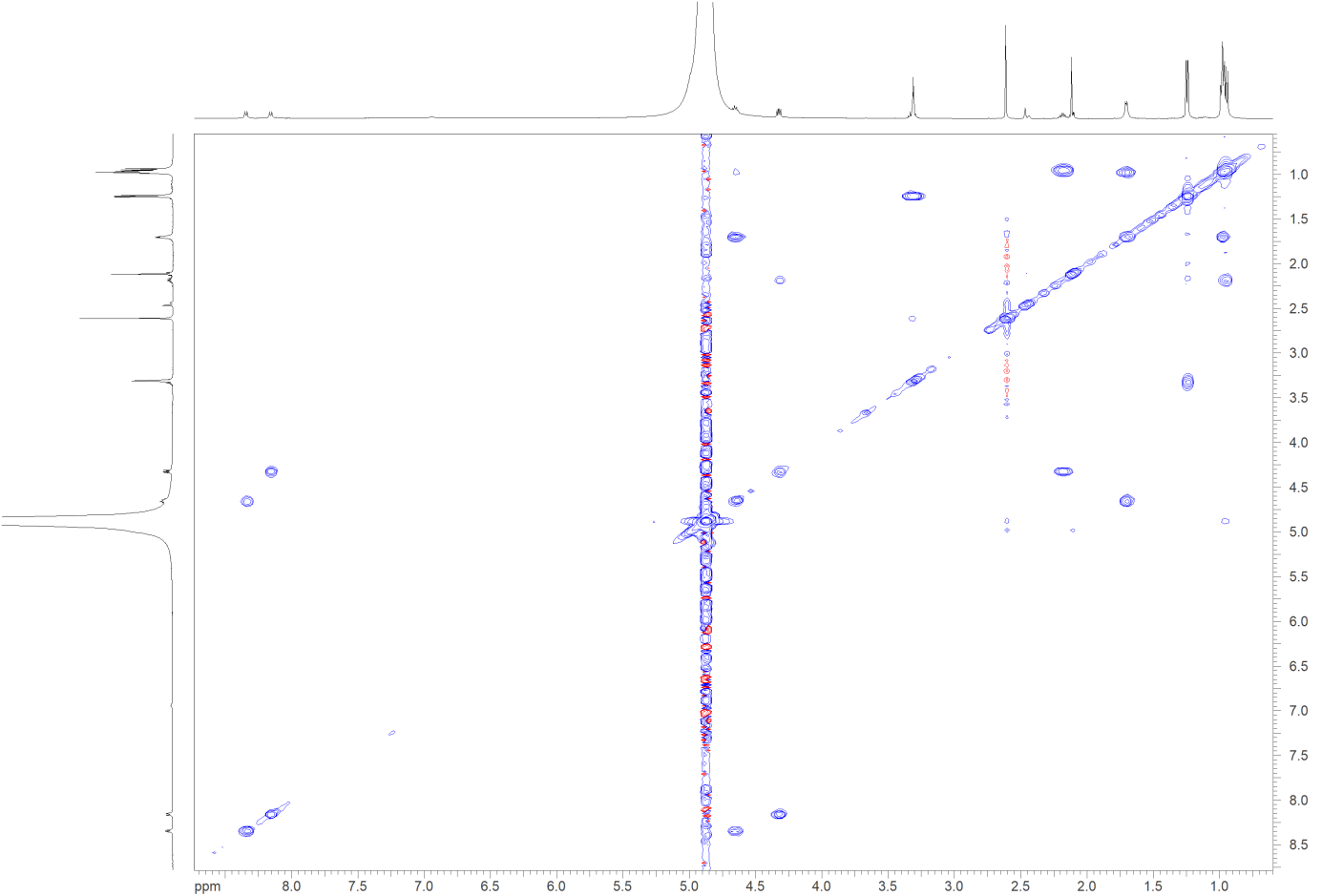


Figure S22: ^1^H-^1^H-COSY spectrum of ichizinone C in MeOD-d_3_.


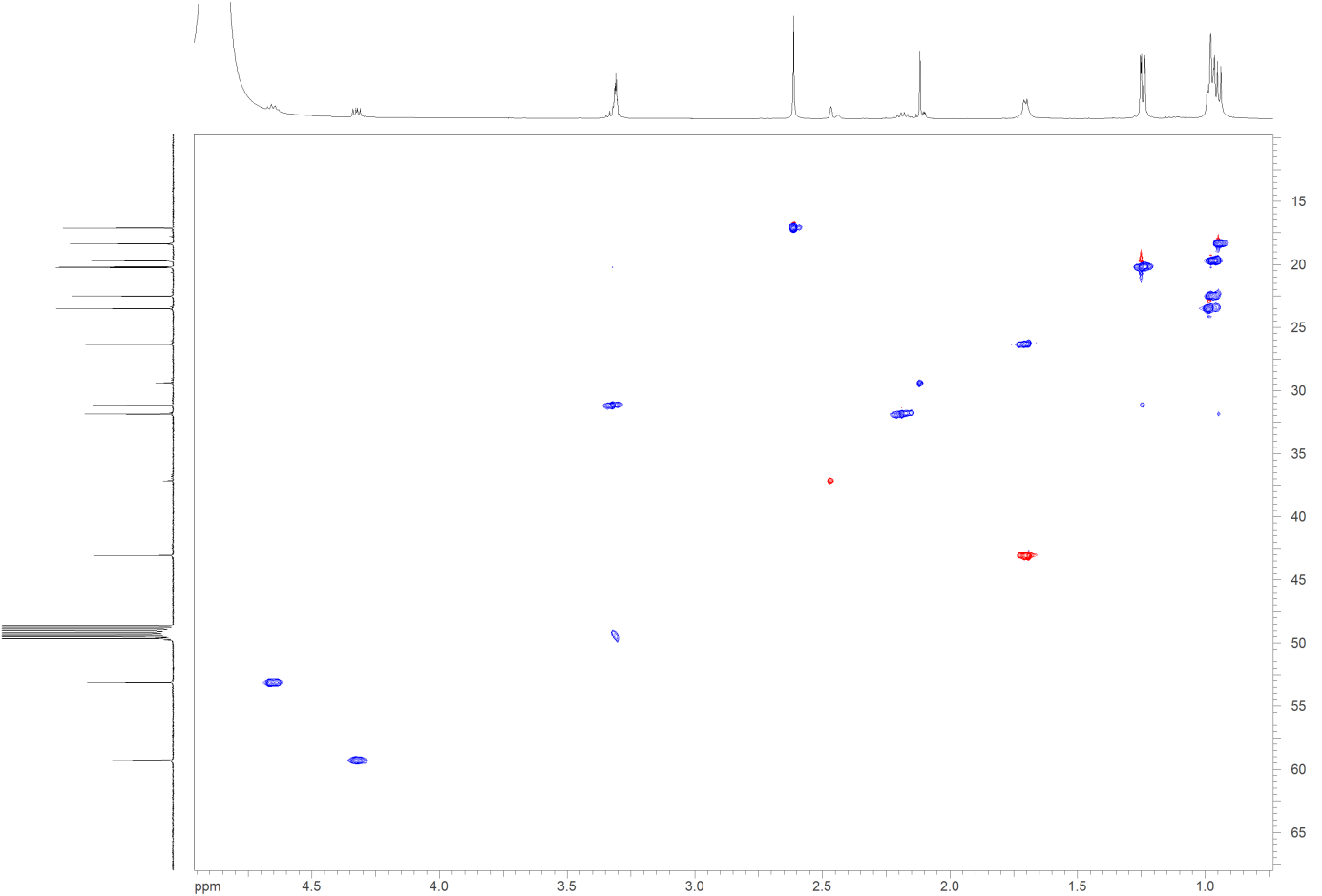


Figure S23: Edited HSQC spectrum of ichizinone C in MeOD-d_3_.


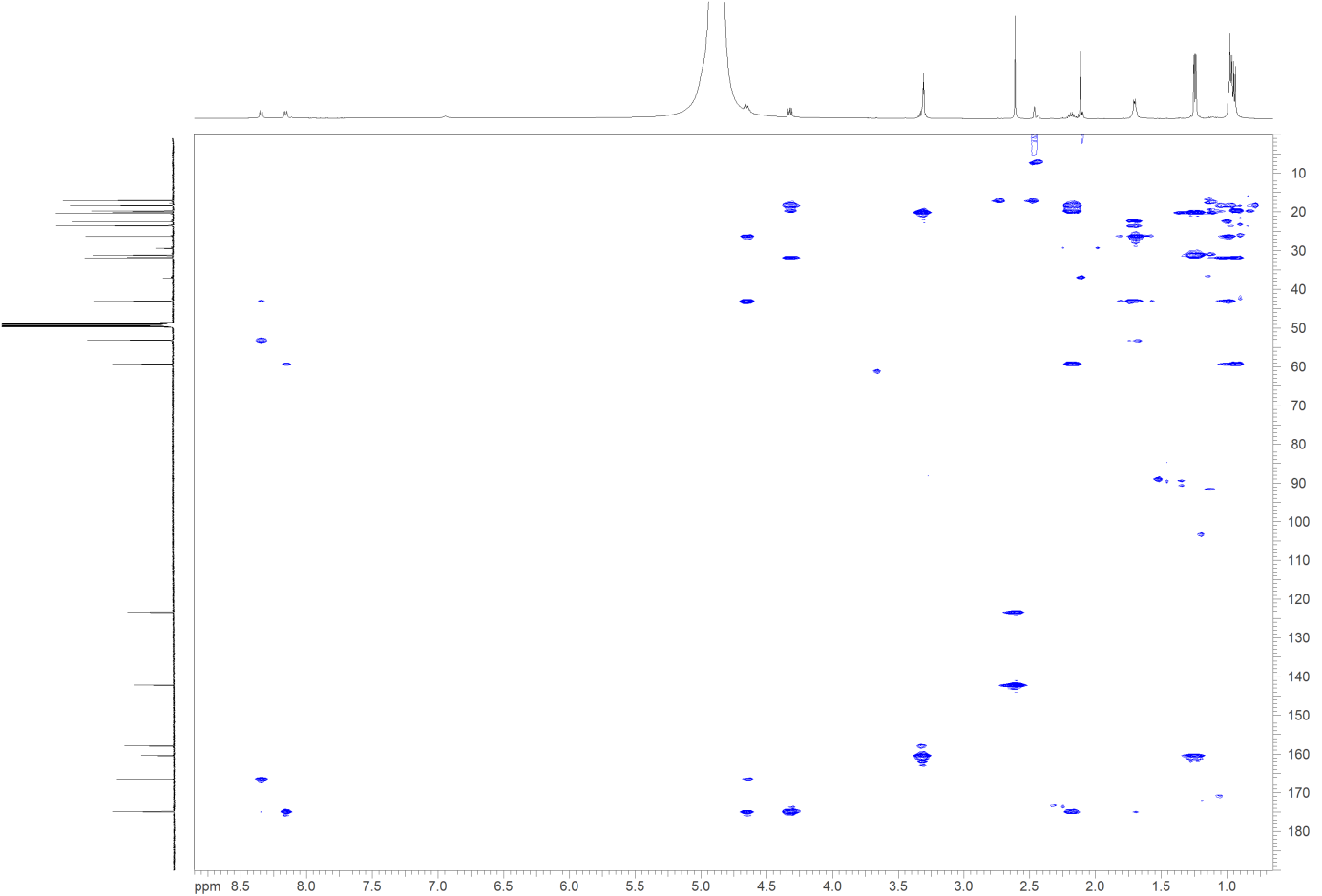


Figure S24: HMBC spectrum of ichizinone C in MeOD-d_3_.


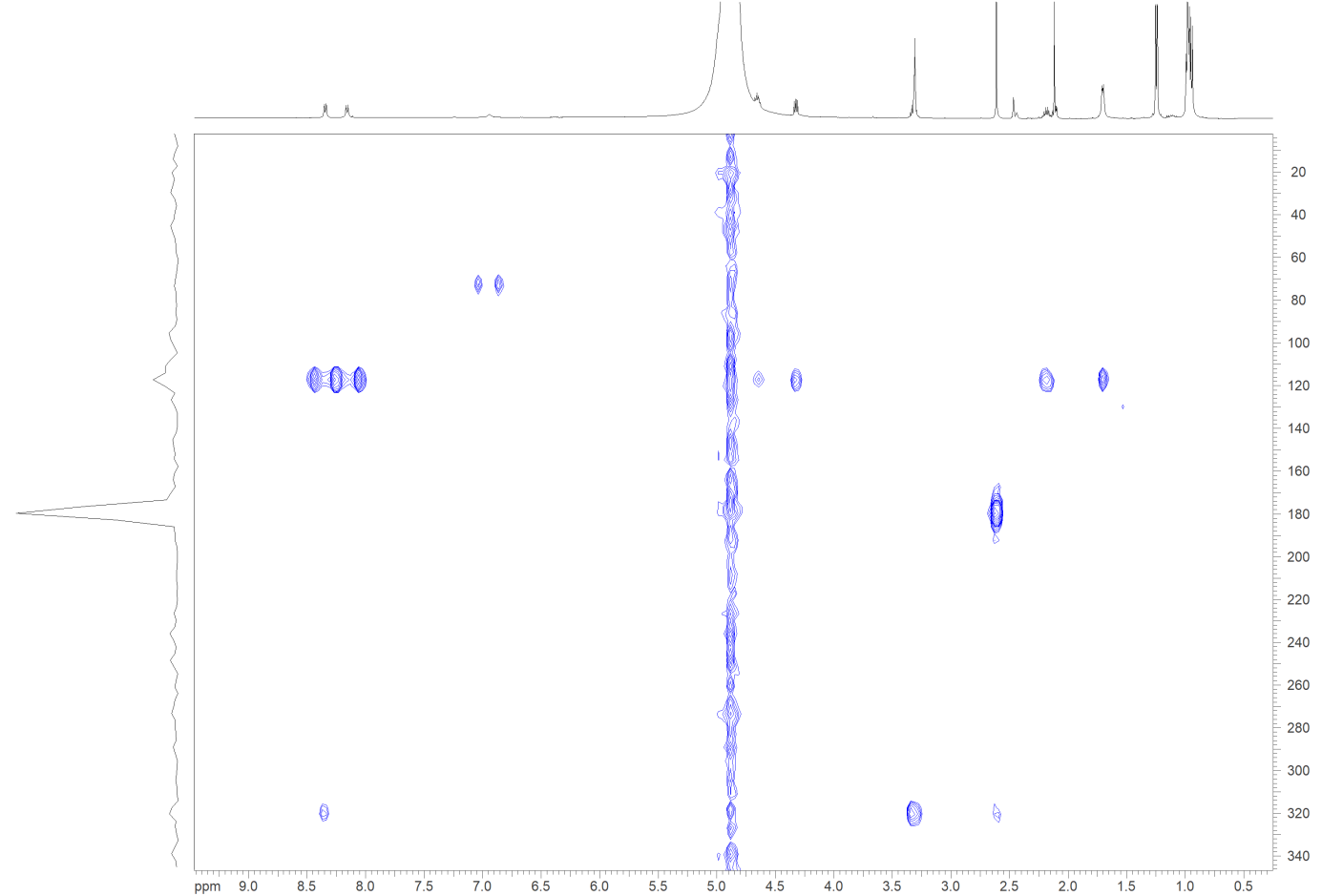


Figure S25: ^15^N-HMBC spectrum of ichizinone C in MeOD-d_3_.

**MS/MS Fragmentation**


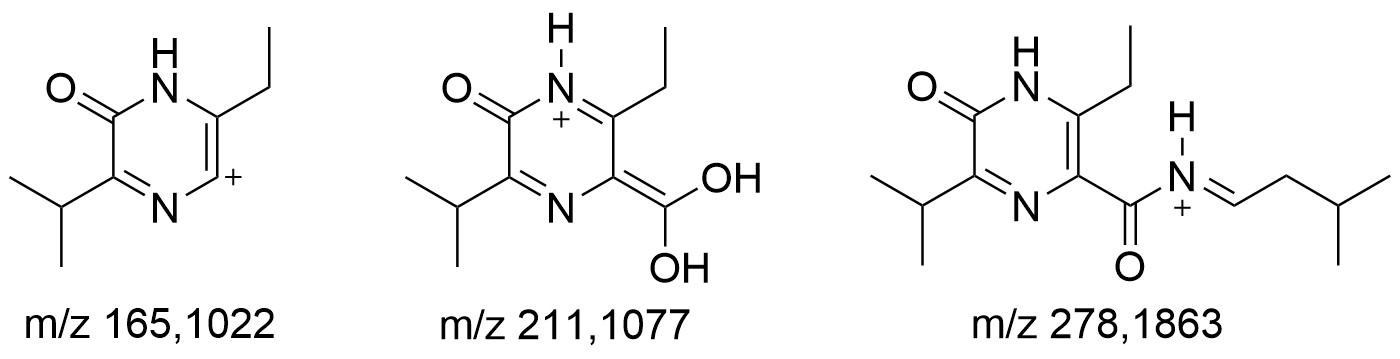


Figure S26: MS-fragmentation spectrum of ichizinone A and the suggested fragments including the calculated monoisotopic mass.


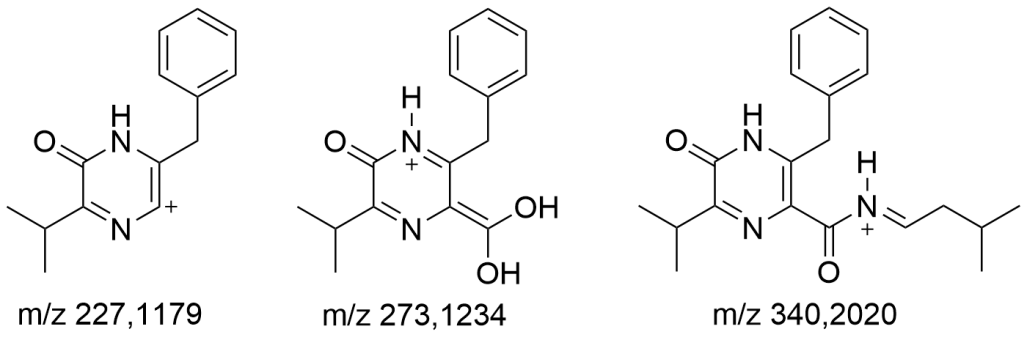


Figure S27: MS-fragmentation spectrum of ichizinone B and the suggested fragments including the calculated monoisotopic mass.

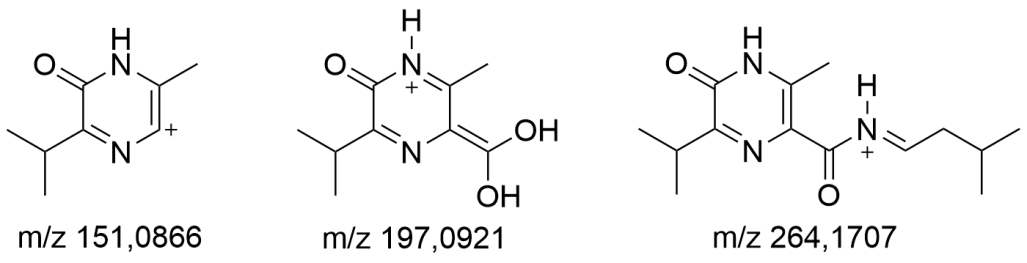


Figure S28: MS-fragmentation spectrum of ichizinone C and the suggested fragments including the calculated monoisotopic mass.

**Marfey’s analysis**

Standards in D- and L-configuration were derivatized with L-FDLA and compared with the amino acids derived from hydrolysis of ichizinone C (fig. S 30)

*
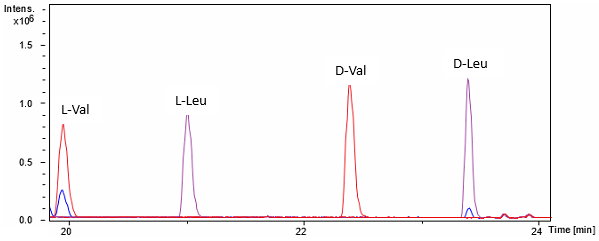
*

Figure S29: Marfey’s chromatograms of the ichizinone hydrolysate (blue) and the amino acids standards (red: val; purple: leu) derivatized with L-FDLA.

References

1. Myronovskyi, M., et al., *Generation of a cluster-free Streptomyces albus chassis strains for improved heterologous expression of secondary metabolite clusters.* Metabolic engineering, 2018. **49**: p. 316-324.

2. Flett, F., V. Mersinias, and C.P. Smith, *High efficiency intergeneric conjugal transfer of plasmid DNA from Escherichia coli to methyl DNA-restricting streptomycetes.* FEMS microbiology letters, 1997. **155**(2): p. 223-229.

3. Grant, S.G., et al., *Differential plasmid rescue from transgenic mouse DNAs into Escherichia coli methylation-restriction mutants.* Proceedings of the National Academy of Sciences, 1990. **87**(12): p. 4645-4649.

4. Myronovskyi, M., et al., *Generation of new compounds through unbalanced transcription of landomycin A cluster.* Applied microbiology and biotechnology, 2016. **100**: p. 9175-9186.
